# Differential roles of human tau isoforms in the modulation of inflammation and development of neuropathology

**DOI:** 10.1016/j.nbd.2025.106942

**Published:** 2025-05-08

**Authors:** Brian Spencer, Aaron Schueler, Daniel Sung, Robert A. Rissman

**Affiliations:** Department of Physiology and Neuroscience, Keck School of Medicine of the University of Southern California, San Diego, CA, USA

**Keywords:** Alzheimer’s disease, Tau, 3 repeat tau, 4 repeat tau, Transgenic mouse

## Abstract

Alzheimer’s disease (AD) is the most common tauopathy characterized by progressive accumulation of Aß and tau neuropathology. Tau is expressed in two major isoforms containing either 3 or 4C-terminal repeats, 3R and 4R. Despite tau isoforms occurring in roughly equimolar ratios in AD, the majority of research focus in developed mouse and *in vitro* models focus only on 4Rtau. To generate a more complete model of AD tauopathy and understand specific tau isoform-mediated neuropathology and neurodegeneration, we generated a transgenic mouse line expressing both 3Rtau and 4Rtau and determined how this impacted the timing and severity of neuropathological and behavioral changes.

**Methods:**

3Rtau-tg and 4Rtau-tg mice were crossed to generate 3R/4Rtau-tg bigenic mice. At 3, 6, and 9 months of age, mice were assessed for behavior, neuropathology and RNA expression.

**Results:**

3R/4Rtau bigenic mice expressed increased tau and phosphorylated tau in the hippocampus and cortex compared to single (3R or 4R) transgenic cohorts as early as 3-months of age and this was accompanied with increased astrogliosis and microglial activation. Bigenic mice had significantly greater behavioral deficits compared to either single transgenic littermates in spatial learning and memory as well as nest building, indicative of depression and/or cognitive deficits.

**Conclusion:**

This new mouse model of tauopathy more completely recapitulates the pattern, severity and accumulation of tau and associated neuropathology and behavioral changes observed in human tauopathies such as AD. 3R/4Rtau-tg bigenic mice should supplant existing single transgenic tau models for general validation of therapeutic targets and investigations of novel therapies on tauopathy endpoints.

## 1. Introduction

Accumulation of tau in neurodegenerative disorders is thought to be mechanistically linked to cell loss and cognitive decline. Tau disorders, referred to as “tauopathies” include Alzheimer’s Disease (AD), Pick’s Disease (PiD) and Fronto-temporal lobar degeneration (FTD) (Dickson et al., 2011). Recent advances in AD therapeutics using antibodies against forms of amyloid-beta (Aß) have shown success in clinical trials (Cummings et al., 2024) and new agents targeting tau are now being tested (Ayalon et al., 2021; Florian et al., 2023; Mummery et al., 2023; Sexton et al., 2022).

AD involves the progressive accumulation of Aß into oligomers and plaques and intra-neuronal tau aggregates, forming neurofibrillary tangles that lead to neuronal synaptic dysfunction and neuronal death (Bachiller et al., 2018; Jack Jr et al., 2019; Sebastian-Serrano et al., 2018; Terry et al., 1994). In fact, tau pathology correlates more directly with dementia than Aß accumulation (Braak and Braak, 1997; Goedert, 2004; Hanseeuw et al., 2019). As the primary tubulin-associated protein in neurons, tau is a major neuronal cytoskeletal protein found in the CNS encoded by the gene MAPT (Del and Iqbal, 2005). Alternative splicing generates two different major forms of tau containing either 3 or 4–32 amino acid repeats (Andreadis et al., 1992). These 3R and 4R tau species are differentially expressed in neurodegenerative diseases with Corticobasal degeneration (CBD) and Progressive supranuclear palsy (PSP) primarily expressing the 4Rtau isoform while PiD primarily expresses the 3Rtau isoform. In contrast, both 3R and 4Rtau isoforms are found accumulating in roughly similar ratio in AD and FTD (Dickson et al., 2011).

Immunotherapy approaches for tau in AD have focused on both active and passive immunization. Active immunization trials in animal models have utilized a peptide of tau which elicits a generalized immune response against all tau species (Congdon et al., 2023; Jang et al., 2024; Pedersen and Sigurdsson, 2015; Valera et al., 2016). Similarly, passive immunization strategies have been developed targeting either total 4R tau protein or phosphorylated or acetylated tau (Congdon et al., 2023; Pedersen and Sigurdsson, 2015; Valera et al., 2016). In fact, tau immunotherapy in amyloid precursor protein (APP)-transgenic (tg) mouse models of AD show reduction in not only total tau, but also Aß oligomers, suggesting that targeting tau may have beneficial downstream effects (Castillo-Carranza et al., 2015; Dai et al., 2017; Levenson et al., 2016). Recently, tau immunotherapy has moved to human clinical trials including the ADAMANT trial which showed reduced plasma phospho-tau 217 following active immunotherapy (Kovacech et al., 2024).

Current immunotherapy strategies target 4Rtau or total tau protein. Because most pre-clinical mouse models of AD involving tau expression overexpress only the 4Rtau protein (Lewis et al., 2000; Ramsden et al., 2005; Yoshiyama et al., 2007), these therapeutics have been successful at this stage. However, AD is characterized by the accumulation of both 4Rtau and 3Rtau which may underlie the reasoning why no tau treatment strategies have been successful to date. We recently developed and characterized a mouse expressing the 3-repeat tau as a model of Pick’s disease/ fronto-temporal dementia (Arner et al., 2018; Rockenstein et al., 2015). For this model we used the 0N3R human tau containing the L266V mutation associated with FTD and the G272V mutation associated with hereditary PiD (Rockenstein et al., 2015). To develop a model that better represents tau accumulation in AD, we crossed our 3Rtau-tg mouse with an established 4-repeat tau mouse line, which expresses the 1N4R human tau containing the P301S mutation associated with early onset FTD (Yoshiyama et al., 2007). Here we demonstrate greater progressive accumulation of tau and phosphorylated tau in the 3R/4Rtau-tg mouse compared to the either 3Rtau-tg or 4Rtau-tg mouse. In addition, we show significantly greater inflammation in the hippocampus and cortex when both 3R and 4Rtau are expressed. Finally, we observed significantly greater behavioral deficits (learning, memory and anxiety) in 3R/4Rtau-tg mice compared to either single transgenic mouse line alone. Thus, we have demonstrated that expression of both major isoforms of tau in the mouse can better recapitulate the tauopathy observed in AD patients and may be a better model to test novel tauopathy therapeutics for AD.

## 2. Materials and methods

### 2.1. Transgenic mouse lines

3Rtau transgenic mice over express human 3 repeat tau (0 N,3R 353) variant L266V and G272V from the mThy-1 promoter on the DBA background (Jackson Laboratories, Cat# 038799, RRID: IMSR_JAX:038799) (Rockenstein et al., 2015). 4Rtau transgenic mice (PS19) over express human 4 repeat tau (1 N,4R 412) variant P301S from the MoPrP promoter on the B6C3H/F1 background (Jackson Laboratories, Cat# 008169, RRID: IMSR_JAX:008169) (Yoshiyama et al., 2007). At least 10 animals (5 M, 5F) for each genotype and age were included in the study. All experimental studies involving animals were approved by Institutional Animal Care and Use Committee of the University of California San Diego and performed in accordance with relevant guidelines and regulations established by NIH Guided for the Care and Use of Laboratory Animals under protocol #S02221.

### 2.2. Behavioral testing

Spatial learning and memory were investigated using the Morris water maze as previously described (Spencer et al., 2016). A pool (diameter 180 cm) was filled with opaque water (24 °C) and mice were first trained to locate a visible platform (days 1–3) and then a submerged hidden platform (days 4–7) in three daily trials 2–3 min apart. Mice that failed to find the hidden platform within 90 s were placed on it for 30 s. The same platform location was used for all sessions and all mice. The starting point at which each mouse was placed into the water was changed randomly between two alternative entry points located at a similar distance from the platform. In addition, on the final day of testing the platform was removed and the time spent by mice in the correct quadrant was measured (Probe test). The duration of the probe test was 40 s. Time to reach the platform (escape latency) was recorded with a Noldus Instruments EthoVision video tracking system (San Diego Instruments, San Diego, CA) set to analyze two samples per second.

Nest building is important for the conservation of warmth and offspring survival and is displayed by both male and female mice (Deacon, 2012). The scoring for nesting is based on a scale of 1–5, with a score of zero meaning the nestlet was not torn at all and a score of five meaning an ideal nest was produced (Arner et al., 2018; Deacon, 2012). This score is based on the amount of the nestlet that was used, as well as the quality of the nest. The percentage of nestlet used was based on weight of the provided nestlet that was torn.

Total activity was measured as previously described (Rockenstein et al., 2015) in a high-density cage rack system from Kinder Smart Frame cage rack (Kinder Scientific, Poway, CA). This system continuously monitors the animal’s location in X, Y, Z coordinate space within the chamber using a 7 × 15 beam configuration. On the day of testing, animals were transported in their home cages to the behavioral testing room. Each individual animal was placed into the test chamber and data collection begins immediately. Test session duration was 10 min and animals were tested on 3 consecutive days followed by a 4th test 2 days later. Beam breaks were quantified for the test period and the 4 test days are averaged.

### 2.3. Immunohistochemistry

Following behavioral analysis, mice were sacrificed following NIH guidelines. All animals were deeply anesthetized with isofluorane and then decapitated. The right hemibrains were post-fixed for 48 h in 4 % phosphate-buffer formaldehyde (pH 7.4) at 4 °C and then sagittal sectioned at 40um (Vibratome 2000; Leica). The left hemibrain was snap frozen in liquid nitrogen and then stored at −80 °C until protein/ RNA analysis.

For neuropathological examination of animals aged 3, 6 and 9 months, serial brain between 1.25 and 1.75 mm lateral to Bregma were immunolabeled overnight with antibodies against 3Rtau (Millipore, Cat# 05–803, RRID: AB_310013), 4Rtau (Millipore, Cat# 05–804, RRID: AB_310014), total tau (Abcam Cat# ab80579, RRID:AB_1603723), P-tau (Ser202,Thr205) (Thermo Fisher Scientific, clone AT8, Cat# MN1020, RRID:AB_223647), NeuN (Millipore Cat# MAB377, RRID:AB_2298772), GFAP (Millipore Cat# MAB3402, RRID:AB_94844) and Iba1 (FUJIFILM Wako Pure Chemical Corporation Cat# 019–19,741, RRID:AB_839504). Sections were then reacted with biotinylated species appropriate secondary antibodies (Vector Laboratories), Avidin D-HRP (ABC Elite; Vector Laboratories) and visualized with diaminobenzidine (DAB). Sections were scanned with NanoZoomer S60 Digital Slide Scanner (Hammamatsu, 20×). Regions of interest were isolated using NDP. View2 and analyzed with CellProfiler or ImageJ. For each group of animals, at least 10 brains were imaged and one image per section from each subregion was analyzed.

### 2.4. Biochemistry

Frozen hemibrains from animals aged 3 and 9 months (*N* = 8 for each group of animals) were sub dissected to generate cortex and hippocampus enriched regions. These were homogenized in homogenization buffer (1× nuclease free PBS, RNAse inhibitor (NEB), protease inhibitor (Mini-Complete, Roche)) with Bead Mill 24 (FisherScientific) using 1.4 mm ceramic beads (FisherScientific). Following homogenization, samples were removed for protein and incubated with 10× RIPA. Protein was quantified by BCA assay (BioRad). For Western blot analysis, 20μg of total cortex or hippocampus protein per lane was loaded in a 15 % Bis-Tris SDS-PAGE gel (15 % Bis-Tris Stain-Free, BioRad). For controls, 300 ng of *in vitro* phosphorylated and 150 ng of unphosphorylated 0N3R tau and 1N4R tau were also loaded. Gels were transferred onto PVDF membranes using semi-dry Trans-Blot Turbo Transfer System (BioRad). Membranes were probed with antibodies against 3Rtau (Millipore, Cat# 05–803, RRID: AB_310013), 4Rtau (Millipore, Cat# 05–804, RRID: AB_310014), total tau (Abcam, clone Tau5, Cat# ab80579, RRID: AB_1603723), P-tau (Ser202, Thr205) (Thermo Fisher, Scientific, clone AT8, Cat# MN1020, RRID:AB_223647), P-tau (Thr181) (Thermo Fisher Scientific, Cat# 701530, RRID: AB_2532491) or P-tau (Ser396, Ser404) (P. Davies Albert Einstein College of Medicine; New York; USA Cat# PHF1, RRID:AB_2315150) overnight. Incubation with primary antibody was followed by species appropriate secondary antibody with horseradish peroxidase (BioRad) and visualized with enhanced chemiluminescence (SuperSignal West Pico PLUS, ThermoScientific). Analysis of Western blots was performed on ChemiDoc Imaging System (BioRad) using actin (Sigma, Cat# A2103, RRID: AB_476694) as normalization. Blots were stripped (Restore Western blot stripping buffer, ThermoScientific) and re-probed successively in the following order: P-tau (Ser396, Ser404), P-tau (Thr181), total tau, actin, and with separate blots for P-tau (Ser202,Thr205) and actin. Individual blots were generated for 3Rtau and 4Rtau in order to specifically identify these bands.

*In vitro* phosphorylation of recombinant tau was performed essentially as described (Abasi et al., 2024). Briefly, 2 μg of recombinant 0N3R or 1N4R human tau (BioLegend) was resuspended in tau phosphorylation buffer (20 mM HEPES pH 7.2, 50 mM NaCl, 0.5 mM TCEP, 4 mM ATP, 1 mM PMSF, 10 mM MgCl_2_, 1× HALT protease inhibitor (Thermo Fisher Scientific) and then 2 μl cAMP-dependent Protein Kinase A (New England Biolabs) and 0.2 μg of GSK3ß kinase (Sigma Aldrich) were added. This was incubated at 30 °C for 16 h and then heat inactivated at 65 °C for 20 min. The phosphorylated tau was then buffer exchanged (20 mM HEPES, 50 mM NaCl, 0.5 mM TCEP) and concentrated with a Microcon Ultracell centrifugal filtration unit (10 kDa cutoff). Phosphorylation was confirmed by Western blot with various anti-phosphorylated tau antibodies.

RNA was extracted from the remaining homogenized sample (*N* = 8 for each group of animals aged 3 and 9 months) using RNAeasy kit (Qiagen) and quantified by spectrophotometry readings. For cDNA synthesis, 500 ng of total RNA was reverse transcribed using iScript gDNA Clear cDNA Synthesis kit (BioRad). Real Time-PCR (RT-PCR) was performed using IDT PrimeTime Gene Expression Master Mix (IDT) with primer/ probe designed for human 0N3R tau [Fwd primer 5’-CAG GAA AGA TCA GGG GGG; Rev. primer 5’-TGC TTC TTC AGC TTT CAG, Probe 5’-CAT GCA CCA AGA CAA GAG GGT GA] and human 1N4R tau [Fwd primer 5’-CAG GAA AGA TCA GGG GGG, Rev. primer 5’-CCT CAG ATC CGT CCT CAG T, Probe 5’-CAT GCA CCA AGA CCA AGA GGG TGA]. Samples were analyzed with a real time PCR system (CFX96 Real Time System, BioRad). The amount of cDNA was calculated by the comparative threshold cycle method and expressed using mouse actin (Mm. PT.39a.22214843.g; IDT) as an internal control and then this was normalized to single transgenic expression (i.e 3Rtau-tg or 4Rtau-tg).

### 2.5. Statistical analysis

All statistical analysis were performed with GraphPad Prism. Group comparisons were made using two-way ANOVA (mixed model) followed by Tukey’s post-hoc test for comparisons. Significance was set at *p* < 0.05 and results are expressed as mean +/− standard error of mean (SEM).

## 3. Results

### 3.1. Tau immunoreactivity in transgenic mice

**Fig. 1.**
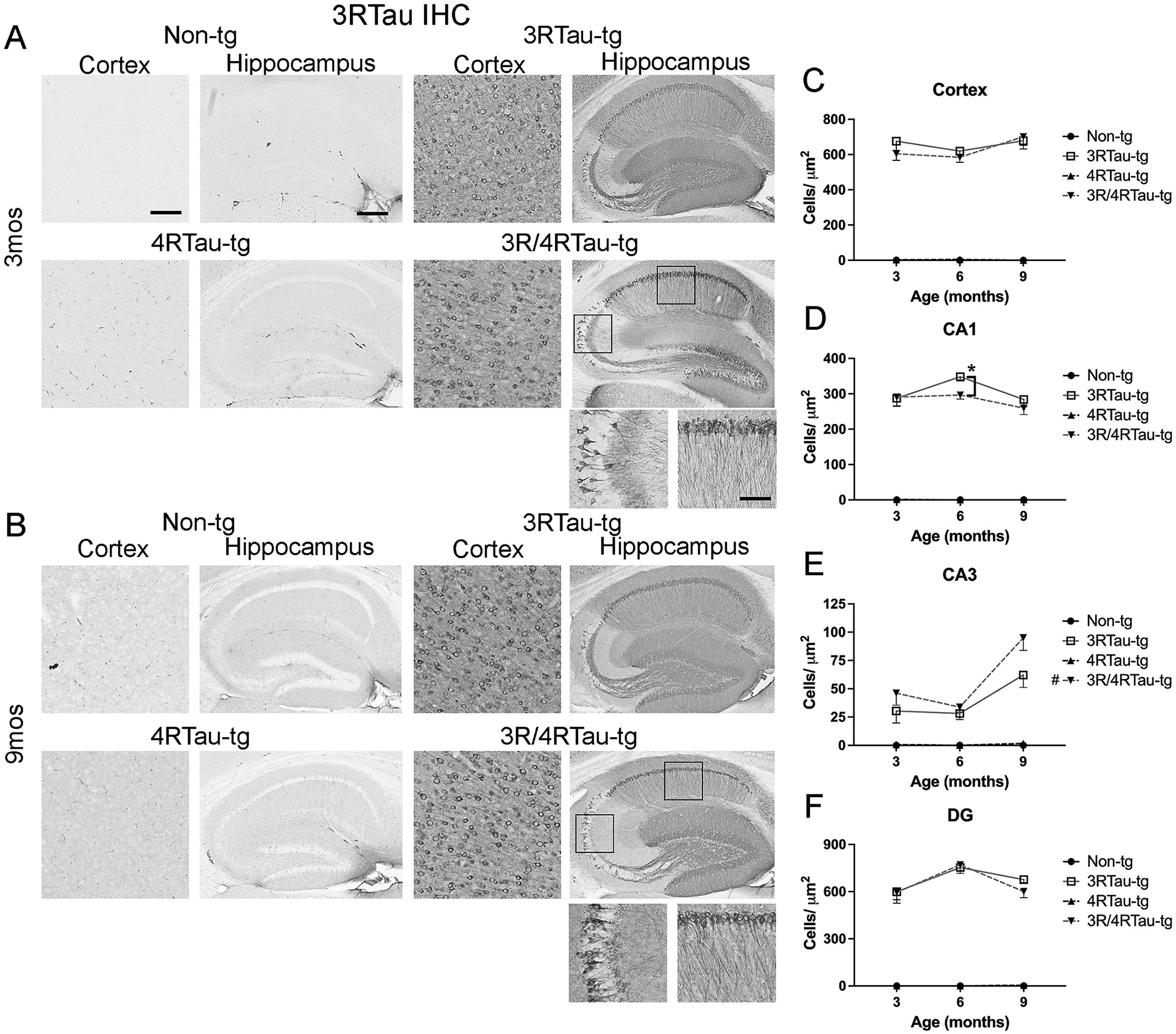
Comparison of patterns of 3Rtau distribution in brains of transgenic mice. Representative sections from (A) 3-month and (B) 9-month-old male non-tg, 3Rtau-tg, 4Rtau-tg and 3R/4Rtau-tg mice were immunostained with an antibody specific for 3Rtau and frontal cortex and hippocampus were analyzed by bright field microscopy. Small panels below the hippocampus are higher magnifications of regions of the CA1 and CA3 indicated by black squares in the lower magnification hippocampus image. Image analysis of numbers of 3Rtau positive cells in the (C) frontal cortex, (D) CA1, (E) CA3 and (F) dentate gyrus (DG) of the hippocampus. Error bars represent +/− SEM. All time points analyzed for 3Rtau-tg and 3R/4Rtau-tg mice were significantly greater than non-tg or 4Rtau-tg (*p* < 0.05). * - indicates significant difference (p < 0.05) between indicated transgenic mice. **#** (figure legend) - indicates significant difference between 3-month and 9-month timepoints (*p* < 0.05). Scale bars = 20 μm in cortex and high-power images of the hippocampus. Scale bar = 200 μm in low power images of hippocampus. *N* = 10 mice per group. Statistical analysis was conducted with two-way ANOVA (mixed model) with Tukey’s test among groups.

To understand longitudinal differences in accumulation of tau in 3Rtau and 4Rtau gene, we performed immunohistochemistry on single and double transgenic mice aged 3, 6 and 9 months (Figs. 1A,B, Supplemental Fig. 7A). 3Rtau and 3R/4Rtau-tg mice expressed 3Rtau in the hippocampus and the cortex at 3-months of age (Fig. 1A). In the hippocampus, expression was observed throughout the CA1 and dentate gyrus, while expression in the CA3 was localized to a few pyramidal neuronal cells of the CA3 as well as interneurons (Fig. 1A). Additionally, there was staining of neurites of the CA1 and supra-pyramidal mossy fibers from the dentate gyrus (Fig. 1A). At 9-months of age, the 3Rtau-tg mice contained significantly more CA1 neurons positive for 3Rtau compared to the 3R/4Rtau-tg mice (Fig. 1B). The overall number of 3Rtau positive cells in the CA3 increased with age, with a significant increase observed in the 3R/4Rtau-tg mice (Fig. 1E). 3Rtau-tg and 3R/4Rtau-tg mice contained similar numbers of 3Rtau positive cells in both the cortex (Fig. 1C) and dentate gyrus of the hippocampus (Fig. 1F). No 3Rtau staining was observed in the hippocampus or cortex of non-tg or 4Rtau-tg mice (Figs. 1A,B, Supplemental Fig. 7A).

**Fig. 2.**
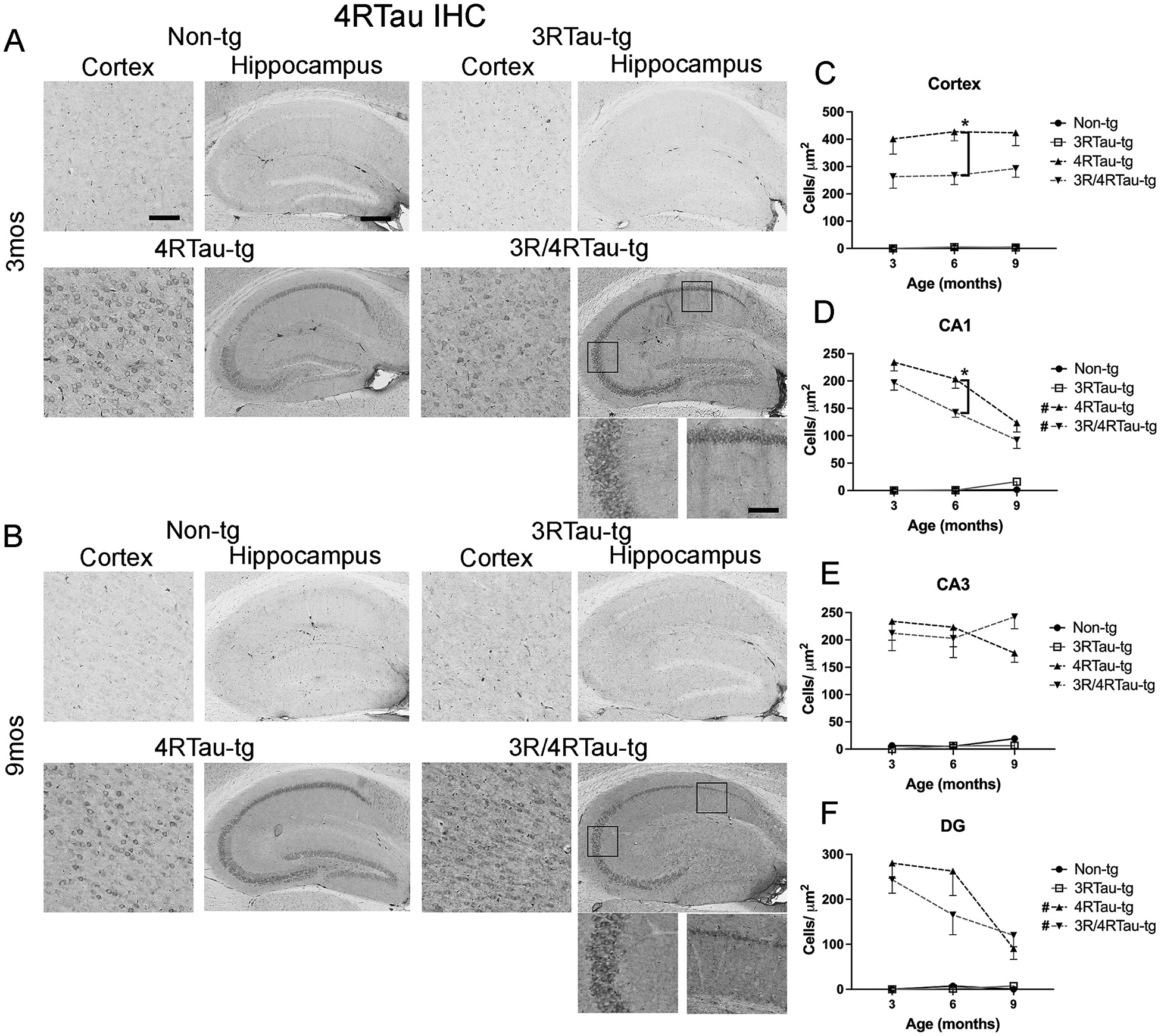
Comparison of patterns of 4Rtau distribution in brains of transgenic mice. Representative sections from (A) 3-month and (B) 9-month-old male non-tg, 3Rtau-tg, 4Rtau-tg and 3R/4Rtau-tg mice were immunostained with an antibody specific for 4Rtau and frontal cortex and hippocampus were analyzed by bright field microscopy. Small panels below the hippocampus are higher magnifications of regions of the CA1 and CA3 indicated by black squares in the lower magnification hippocampus image. Image analysis of numbers of 4Rtau positive cells in the (C) frontal cortex, (D) CA1, (E) CA3 and (F) dentate gyrus (DG) of the hippocampus. Error bars represent +/− SEM. All time points analyzed for 4Rtau-tg and 3R/4Rtau-tg mice were significantly greater than non-tg or 4Rtau-tg (*p* < 0.05). * - indicates significant difference (p < 0.05) between indicated transgenic mice. **#** (figure legend) - indicates significant difference between 3-month and 9-month timepoints (p < 0.05). Scale bars = 20 μm in cortex and high-power images of the hippocampus. Scale bar = 200 μm in low power images of hippocampus. *N* = 10 mice per group. Statistical analysis was conducted with two-way ANOVA (mixed model) with Tukey’s test among groups.

As expected, 4Rtau staining was observed only in 4Rtau-tg and 3R/4Rtau-tg mice (Figs. 2A,B, Supplemental Fig. 7B). Cortical staining was greater in 4Rtau single transgenic compared to the 3R/4Rtau bigenic mouse (Fig. 2C). Similarly, more CA1 neurons were positive for 4Rtau in the single transgenic mouse compared to the bigenic mouse (Fig. 2D). Interestingly, the number of CA1 and dentate gyrus positive neurons were fewer in 9-month mice compared to 3-month mice (Fig. 2D, F) possibly a result of neuronal loss. In contrast to 3Rtau staining, 4Rtau did not appear to accumulate in the neurites or the mossy fibers of the hippocampus. 4Rtau-tg and 3R/4Rtau-tg mice contained similar numbers of 4Rtau positive cells in the CA3 of the hippocampus (Fig. 2E).

**Fig. 3.**
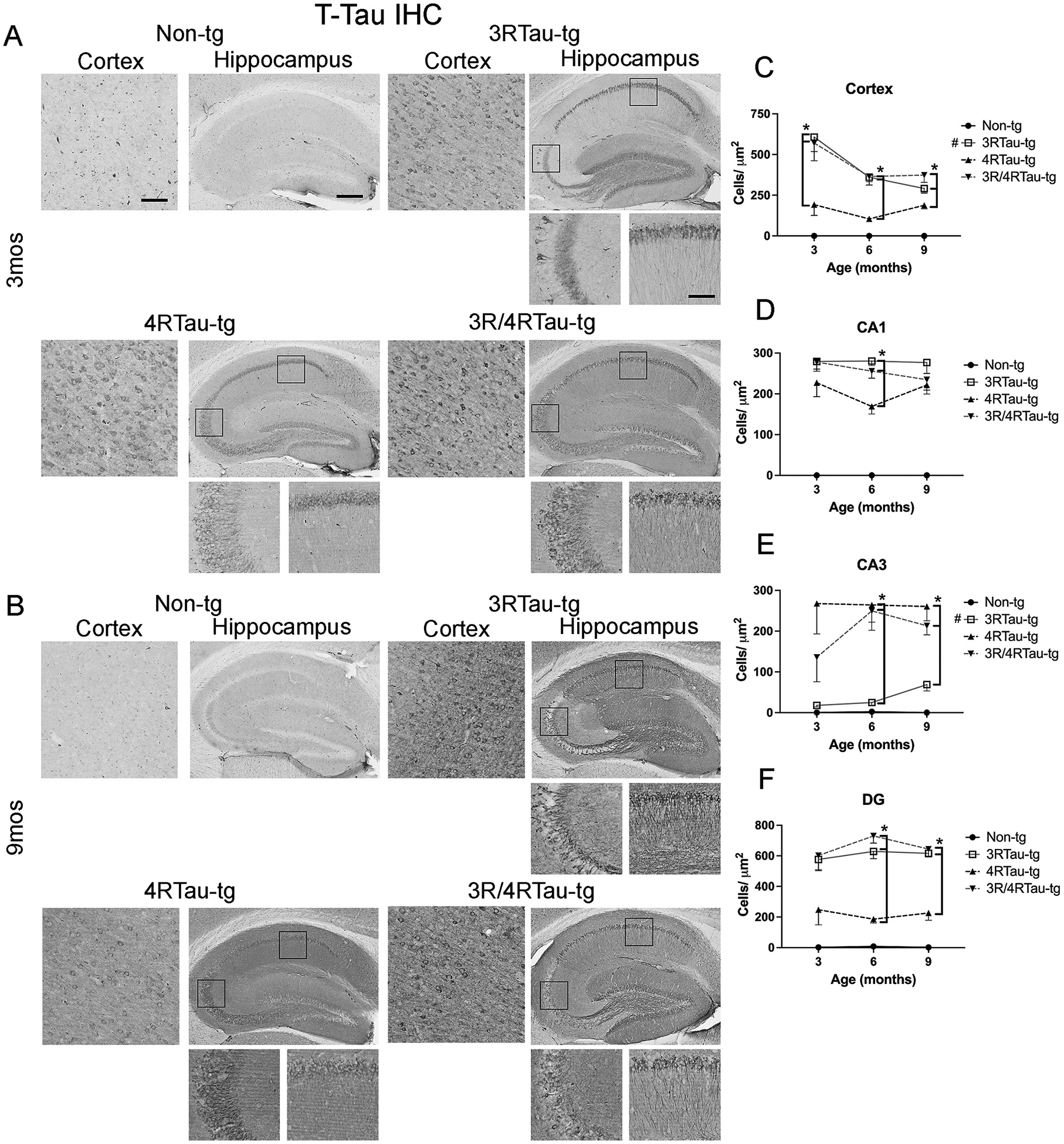
Comparison of patterns of T-tau distribution in brains of transgenic mice. Representative sections from (A) 3-month and (B) 9-month-old male non-tg, 3Rtau-tg, 4Rtau-tg and 3R/4Rtau-tg mice were immunostained with an antibody specific for T-tau and frontal cortex and hippocampus were analyzed by bright field microscopy. Small panels below the hippocampus are higher magnifications of regions of the CA1 and CA3 indicated by black squares in the lower magnification hippocampus image. Image analysis of numbers of T-tau positive cells in the (C) frontal cortex, (D) CA1, (E) CA3 and (F) dentate gyrus (DG) of the hippocampus. Error bars represent +/− SEM. All time points analyzed for 3Rtau-tg, 4Rtau-tg and 3R/4Rtau-tg mice were significantly greater than non-tg (p < 0.05). * - indicates significant difference (p < 0.05) between indicated transgenic mice. **#** (figure legend) - indicates significant difference between 3-month and 9-month timepoints (p < 0.05). Scale bars = 20 μm in cortex and high-power images of the hippocampus. Scale bar = 200 μm in low power images of hippocampus. N = 10 mice per group. Statistical analysis was conducted with two-way ANOVA (mixed model) with Tukey’s test among groups.

**Fig. 4.**
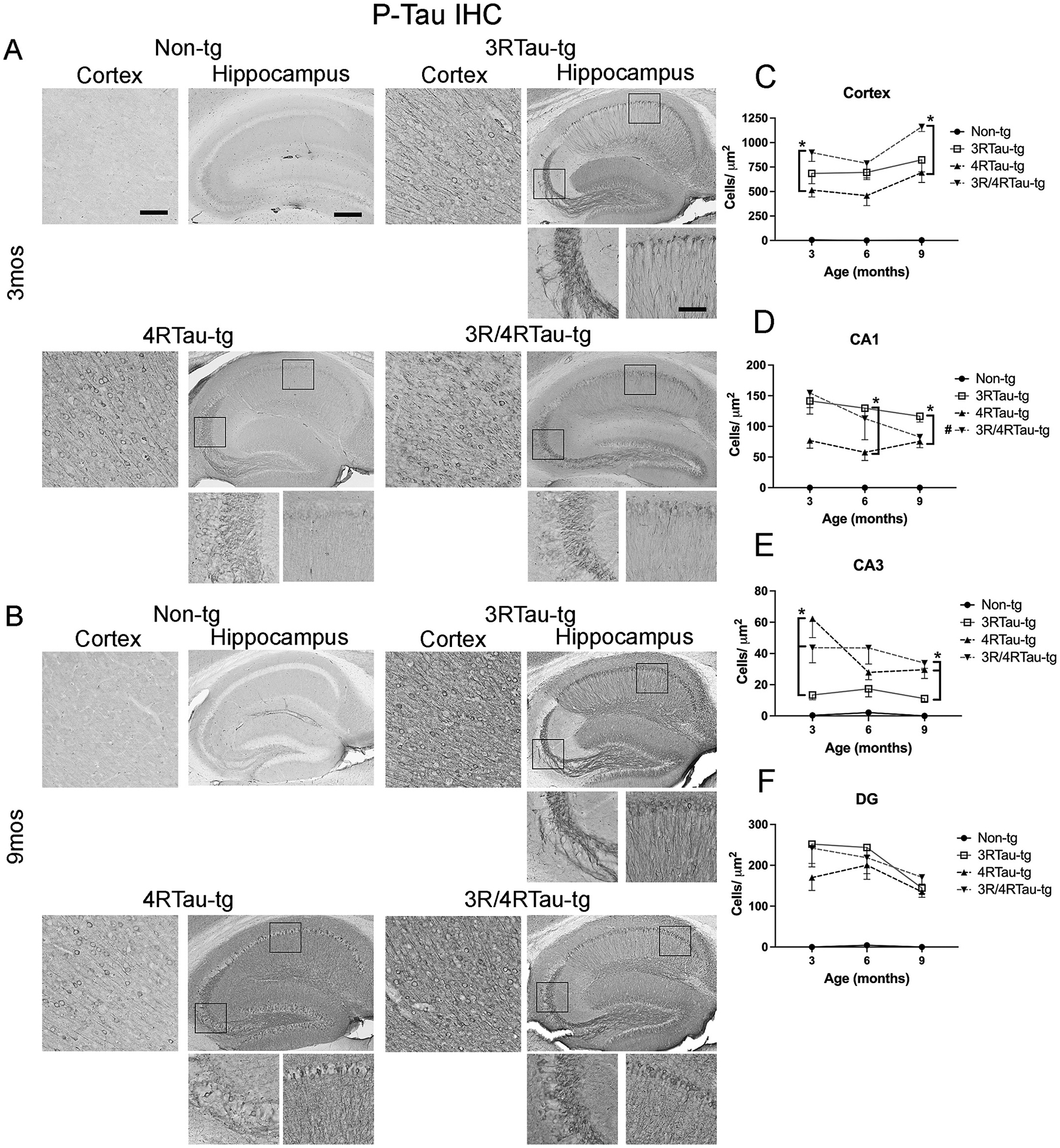
Comparison of patterns of P-tau distribution in brains of transgenic mice. Representative vibratome sections from (A) 3-month and (B) 9-month-old male non-tg, 3Rtau-tg, 4Rtau-tg and 3R/4Rtau-tg mice were immunostained with an antibody specific for P-tau (Ser202/Thr205, AT8) and frontal cortex and hippocampus were analyzed by bright field microscopy. Small panels below the hippocampus are higher magnifications of regions of the CA1 and CA3 indicated by black squares in the lower magnification hippocampus image. Image analysis of numbers of P-tau positive cells in the (C) frontal cortex, (D) CA1, (E) CA3 and (F) dentate gyrus (DG) of the hippocampus. Error bars represent +/− SEM. All time points analyzed for 3Rtau-tg, 4Rtau-tg and 3R/4Rtau-tg mice were significantly greater than non-tg (*p* < 0.05) * - indicates significant difference (p < 0.05) between indicated transgenic mice. **#** (figure legend) - indicates significant difference between 3-month and 9-month timepoints (p < 0.05). Scale bars = 20 μm in cortex and high-power images of the hippocampus. Scale bar = 200 μm in low power images of hippocampus. *N* = 10 or more mice per group. Statistical analysis was conducted with two-way ANOVA (mixed model) with Tukey’s test among groups.

Next, we examined the accumulation of total tau (T-tau) (Figs. 3A, B)) and the phosphorylated tau (P-tau) (Figs. 4A, B). Phosphorylation of tau at Ser202/Thr205 (P-tau) has been demonstrated to lead to self-aggregation and polymerization (Hoover et al., 2010). Total tau and P-tau accumulation expressed similar expression patterns throughout the brains of all tau mice (Figs. 3,4, Supplemental Fig. 7 C,D). Both T-tau and P-tau accumulated in both the cortex and hippocampus with significantly greater accumulation in the cortex (Fig. 3C, 4C), CA1 (Fig. 3D, 4D) and dentate gyrus (Fig. 3F, 4F) in the 3R/4Rtau-tg and 3Rtau-tg mouse compared to the 4Rtau-tg mouse. No significant differences were observed in these areas between the 3Rtau and bigenic mice. 4Rtau-tg mice consistently contained lower numbers of T-tau positive cells than either the single or bigenic mice in the cortex, CA1, and dentate gyrus. In contrast, CA3 of the hippocampus showed significantly fewer positive cells for the 3Rtau-tg mouse compared to the 4Rtau-tg or 3R/4Rtau-tg mice mimicking the pattern of low 3Rtau expression in the CA3 compared to other areas of the hippocampus (Fig. 3E, 4E). The total accumulation of T-tau and P-tau was not significantly greater than a single tg mouse except in the cortex where greater P-tau accumulation was observed compared to either single tg mouse. Staining with ThioS for tau tangles did not show any evidence of tangle formation in 3Rtau-tg, 4Rtau-tg or 3R/4Rtau-tg mice in any area examined suggesting that this mouse model only has tangle-like accumulation of tau (data not shown).

### 3.2. Neurodegenerative pathology in tau transgenic mice

**Fig. 5.**
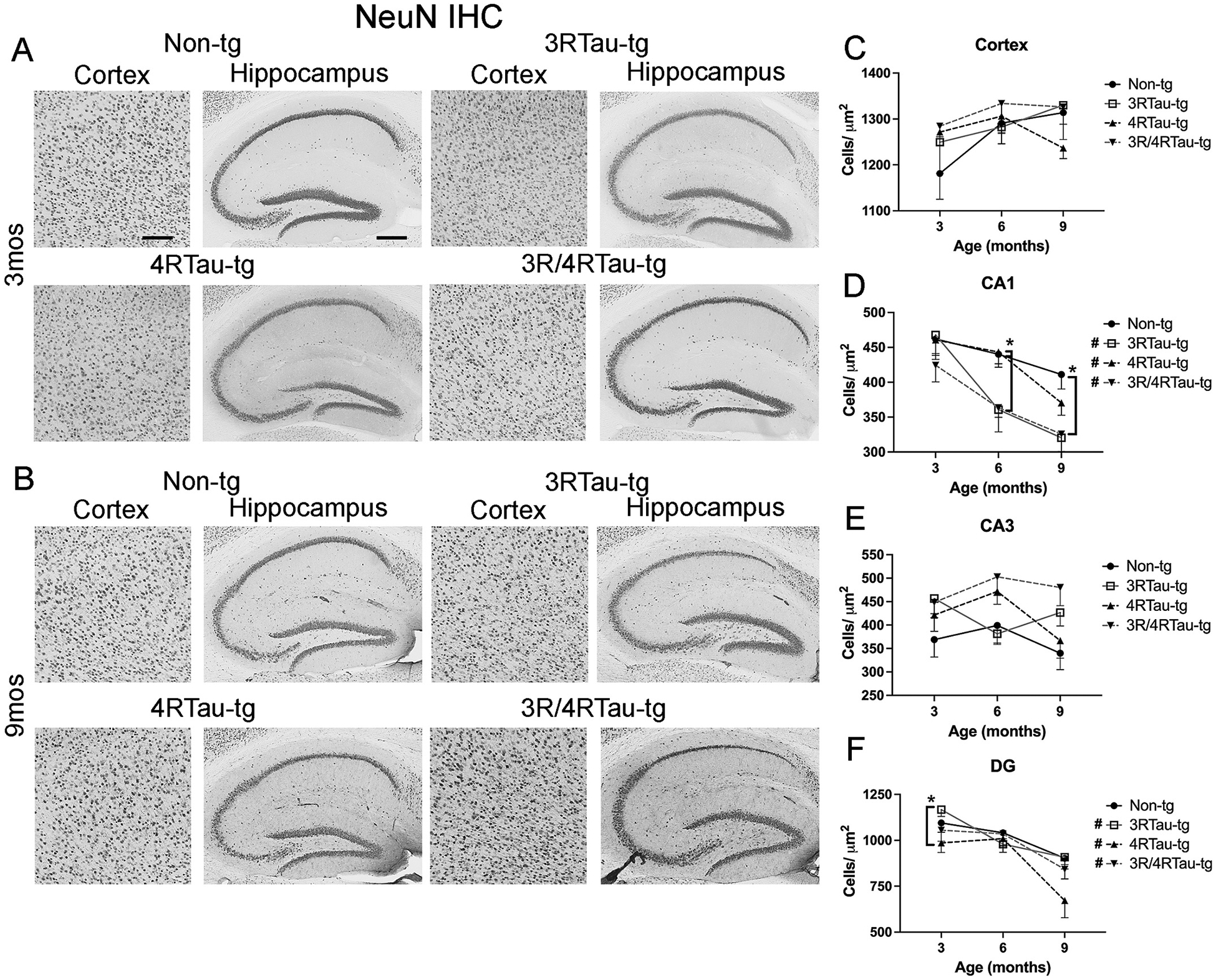
Comparison of patterns of NeuN distribution in brains of transgenic mice. Representative sections from (A) 3-month and (B) 9-month-old male non-tg, 3Rtau-tg, 4Rtau-tg and 3R/4Rtau-tg mice were immunostained with an antibody specific for NeuN and frontal cortex and hippocampus were analyzed by bright field microscopy. Image analysis of numbers of NeuN positive cells in the (C) frontal cortex, (D) CA1, (E) CA3 and (F) dentate gyrus (DG) of the hippocampus. Error bars represent +/− SEM. * - indicates significant difference (p < 0.05) between indicated transgenic mice. **#** (figure legend) - indicates significant difference between 3-month and 9-month timepoints (p < 0.05). Scale bars = 40 μm in cortex and 200um in the hippocampus. N = 10 or more mice per group. Statistical analysis was conducted with two-way ANOVA (mixed model) with Tukey’s test among groups.

To determine whether the decreased number of tau positive cells in the hippocampus was due to neuron loss or reduced tau expression over time, we stained sections for the neuronal marker NeuN. Compared to non-tg mice, 3Rtau-tg and 3R/4Rtau-tg mice showed significant loss of neurons in the CA1 at 6 and 9-months of age (Fig. 5A,B,D, Supplemental Fig. 8A). Over the time period examined, all tau expressing mice showed a significant reduction in neuronal cells in the CA1 and DG compared to non-tg mice (Fig. 5D, F) similar to the loss of 4Rtau staining observed in the same region, suggesting that tau in this region may be mechanistically involved in neuronal loss in this region. In contrast to the loss of T-tau staining in the cortex, no significant loss of cortical neurons was observed among the different mouse lines (Fig. 5C). Although tau expression in the transgenic mice is observed in the dentate gyrus, we did not observe a loss of neurons through NeuN staining (Fig. 5E).

**Fig. 6.**
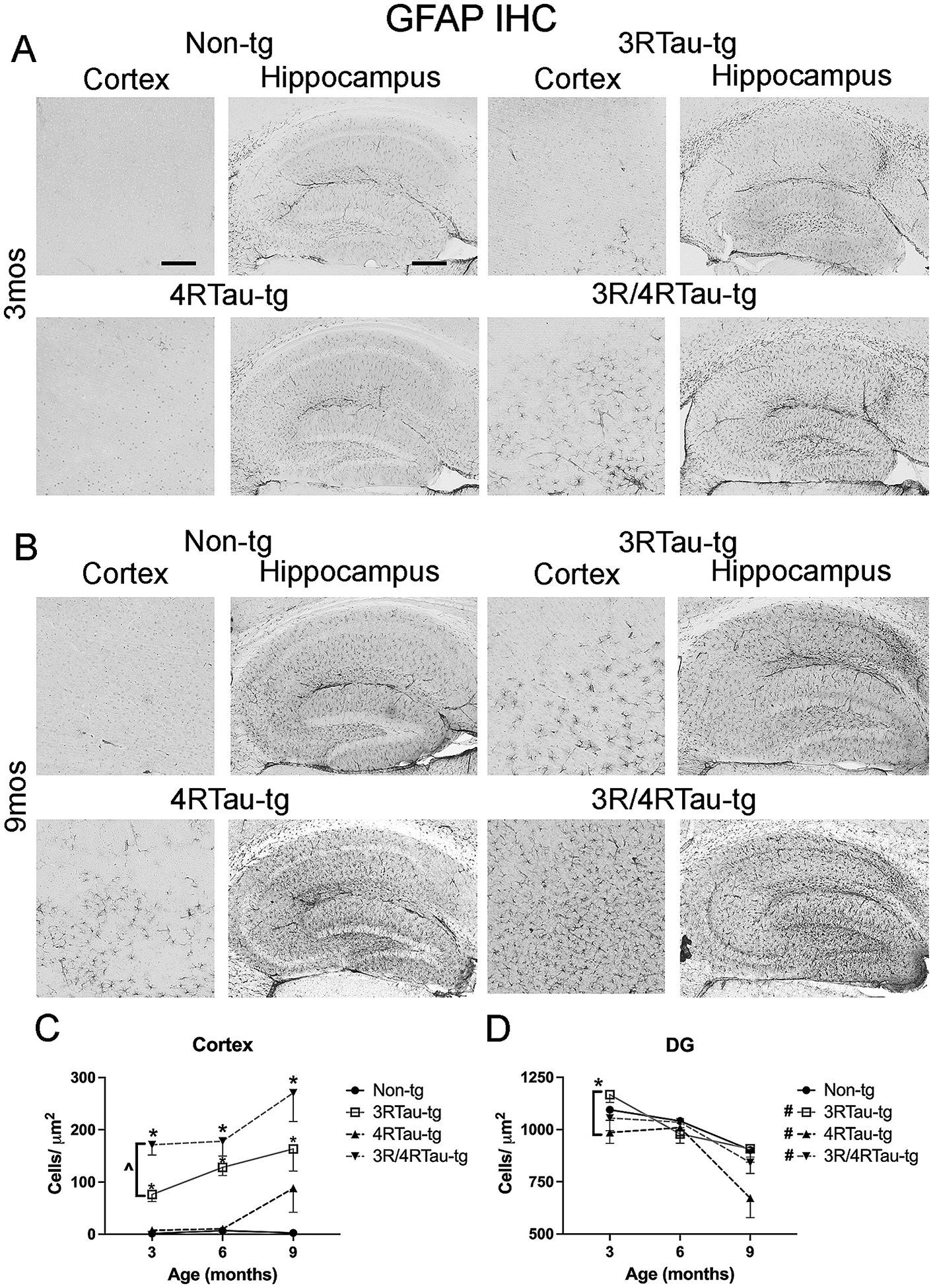
Comparison of patterns of GFAP distribution in brains of transgenic mice. Representative sections from (A) 3-month and (B) 9-month-old male non-tg, 3Rtau-tg, 4Rtau-tg and 3R/4Rtau-tg mice were immunostained with an antibody specific for GFAP and frontal cortex and hippocampus were analyzed by bright field microscopy. Image analysis of numbers of GFAP positive cells in the (C) frontal cortex and (D) hippocampus. Error bars represent +/− SEM. * - indicates significant difference (p < 0.05) between indicated transgenic mice and non-tg mice. ^ - indicated significant difference between indicated groups. **#** (figure legend) - indicates significant difference between 3-month and 9-month timepoints (p < 0.05). Scale bars = 40 μm in cortex and 200um in the hippocampus. N = 10 or more mice per group. Statistical analysis was conducted with two-way ANOVA (mixed model) with Tukey’s test among groups.

Neuroinflammation is a hallmark of AD and includes the proliferation of astrocytes as well as the activation of microglia cells (Alster et al., 2020; Amin et al., 2020; Bachiller et al., 2018; Guzman-Martinez et al., 2019; Lindstrom et al., 2017). We examined the effect of overexpression of tau on the number of astrocytes in the cortex and hippocampus by staining for GFAP (astrocytic marker) (Fig. 6, Supplemental Fig. 8B). At all timepoints, 3Rtau-tg and 3R/4Rtau-tg mice exhibited increased astrocytosis in the cortex compared to non-tg and 4Rtau-tg mice (Fig. 6C). At the earliest time point (3-month), astrocytosis was greater in 3R/4Rtau-tg mice compared to 3Rtau-tg mice (Fig. 6A). At 9-months, an increase in astrocytosis was observed in 4Rtau-tg mice similar to 3Rtau-tg or 3R/4Rtau-tg (Fig. 6B). In the hippocampus, 3R/4Rtau-tg mice had significantly greater astrocytosis compared to non-tg and 4Rtau-tg mice at 3-months of age (Fig. 6D). At 6-months of age, all 3 tau overexpressing mouse lines exhibited an increased astrocytosis compared to non-tg mice (Supplemental Fig. 8B). Elevated astrocytosis in the hippocampus diminished by 9-months of age.

**Fig. 7.**
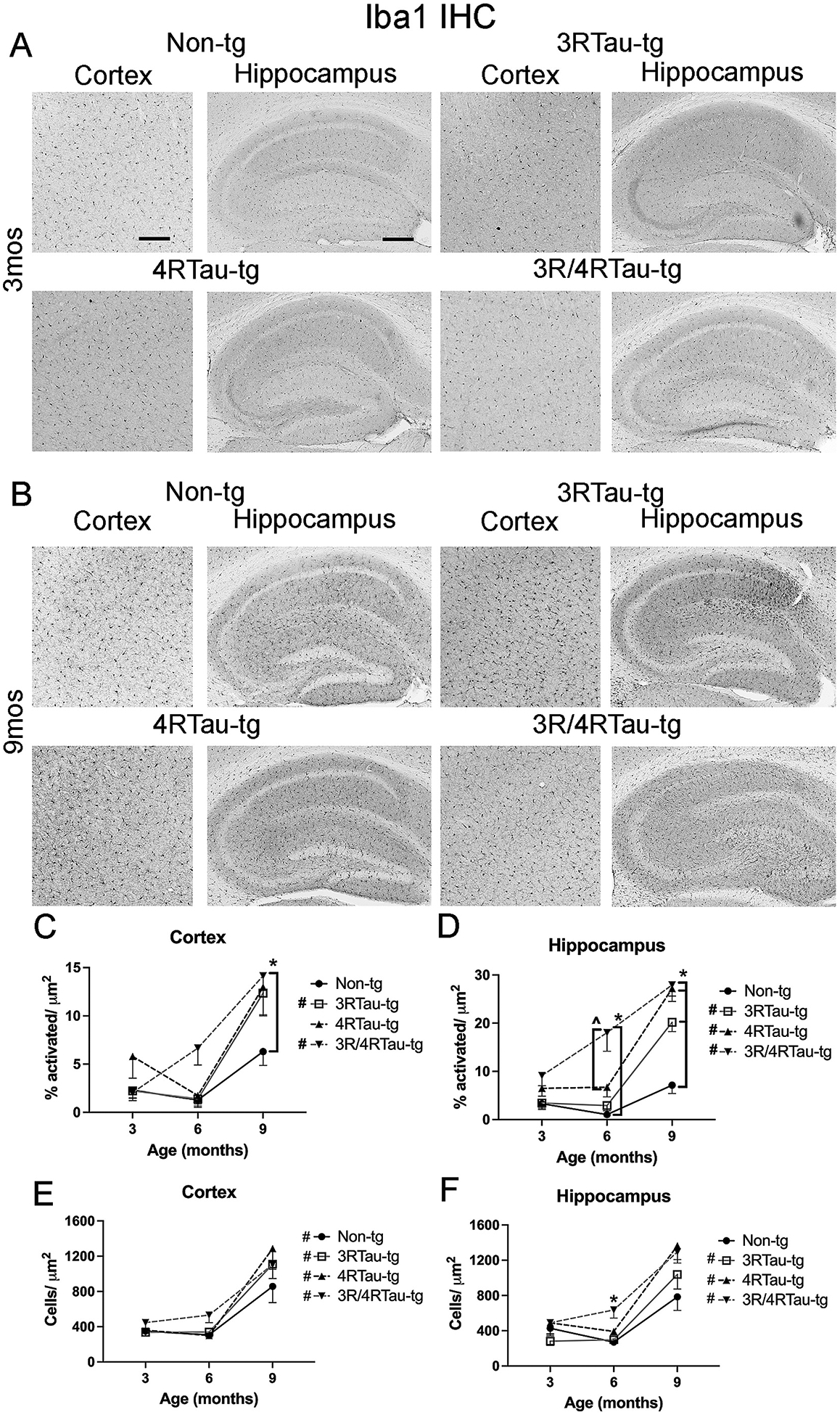
Comparison of patterns of Iba1 distribution in brains of transgenic mice. Representative sections from (A) 3-month and (B) 9-month-old male non-tg, 3Rtau-tg, 4Rtau-tg and 3R/4Rtau-tg mice were immunostained with an antibody specific for Iba1 and frontal cortex and hippocampus were analyzed by bright field microscopy. Image analysis of percentage of activated/ resting Iba1 positive microglial cells in the (C) frontal cortex and (D) hippocampus. Image analysis of total number of Iba1 positive cells in the (E) frontal cortex and (F) hippocampus. Error bars represent +/− SEM. * - indicates significant difference (p < 0.05) between indicated transgenic mice and non-tg mice. ^ - indicated significant difference between indicated groups. **#** (figure legend) - indicates significant difference between 3mos and 9mos timepoints (p < 0.05). Scale bars = 40 μm in cortex and 200um in the hippocampus. N = 10 or more mice per group. Statistical analysis was conducted with two-way ANOVA (mixed model) with Tukey’s test among groups.

In contrast to the changes observed in astrocytes at early time points, significant differences in microglia as measured through Iba1 immunostaining manifested only at later time points (Fig. 7A,B, Supplemental Fig. 8C). Quantification of activated microglia at 9-months, determined by increased size and length of processes, indicated increased microglia activation in all 3 tau overexpressing mouse lines (Fig. 7C, D). While only the 3R/4Rtau-tg mice showed statistically greater activation compared to non-tg at 9-months, both the 3Rtau-tg and 3R/4Rtau-tg mice showed a significant increase in activated microglia between 3 and 9-months of age. Similar results were observed in the hippocampus, where increased activated microglia were observed for all 3 tau over-expressing mouse lines. At 6-months of age, significantly greater activated microglia were observed in the hippocampus of 3R/4Rtau-tg mouse compared to non-tg mice, whereas there was no significant increase in the cortex. The total number of microglia increased from 3 to 9-months in the cortex and hippocampus of all the mouse lines in the study (Fig. 7E,F).

### 3.3. Characterization of tau mRNA and protein expression in tau transgenic mice

**Fig. 8.**
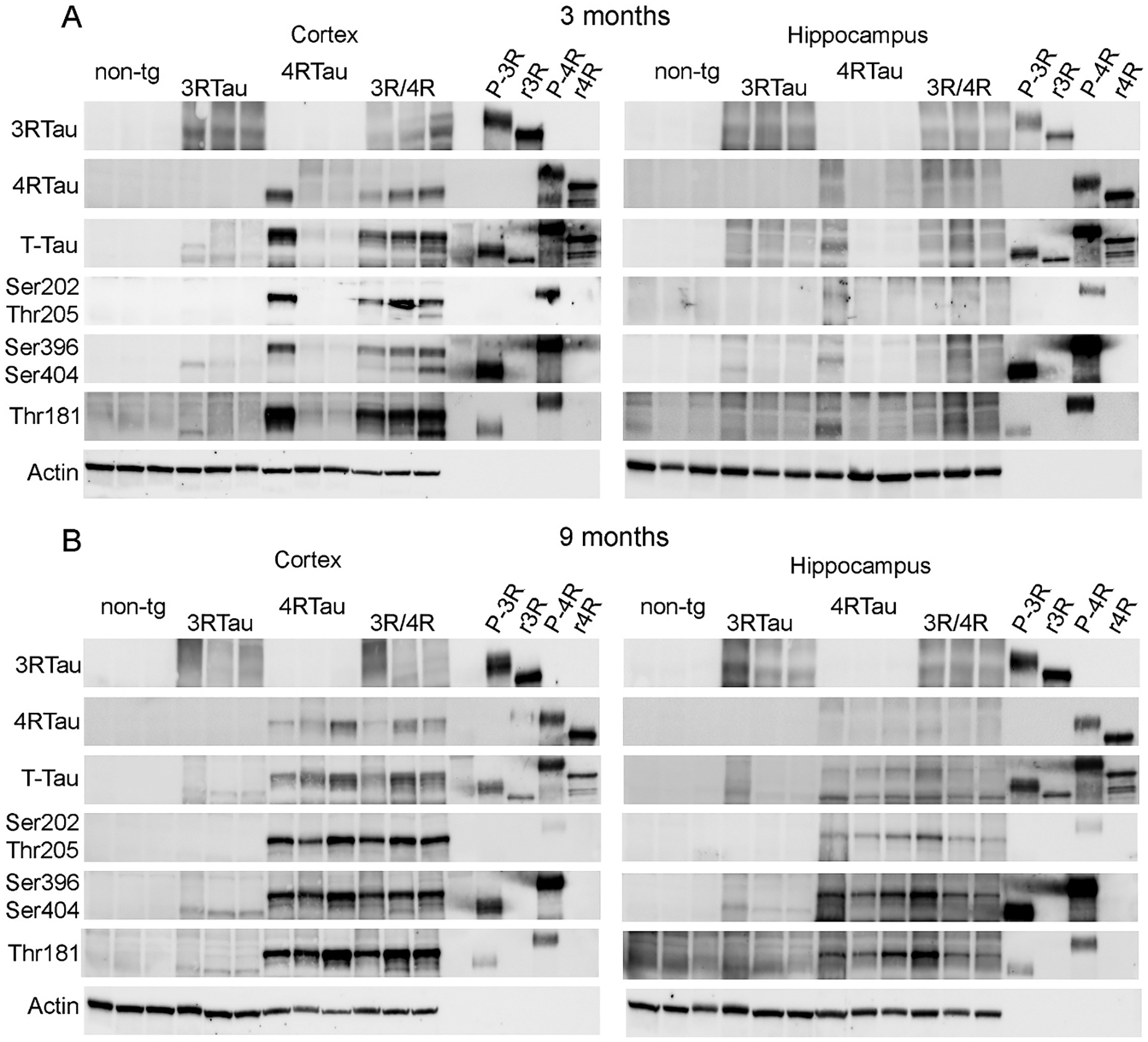
Accumulation of tau in the brains of transgenic mice. Frozen hemibrains were sub-dissected to isolate cortical and hippocampal regions. Total protein was isolated for analysis. Representative Western blots from (A) 3-month and (B) 9-month-old mice analyzed with antibodies specific for 3Rtau, 4Rtau, T-tau, P-tau (Ser202/Thr205), P-tau (Ser396/Ser404), P-tau (Thr181) and actin. Recombinant 0N3R (r3R) and 1N4R (r4R) human tau and *in vitro* phosphorylated 0N3R (P-3R) and 1N4R (P-4R) tau are included to compare size of proteins from mouse brain samples.

To better characterize tau in transgenic mice, we examined frozen hemibrains from cohorts of mice at 3-month and 9-month age groups. Brain regions enriched for cortex or hippocampus were homogenized and assayed for protein accumulation by Western blot. Because tau antibodies recognize numerous bands on a Western blot, we added controls of recombinant 0N3R and 1N4R tau the same as expressed by the 3Rtau-tg and 4Rtau-tg mice respectively as well as controls of *in vitro* phosphorylated 0N3R and 1N4R tau. Previous characterization of the *in vitro* phosphorylation of a 2N4R tau revealed phosphorylation at Thr181, Ser202, Thr205, Thr212, Thr212 and Ser214, S396 and S404 (Abasi et al., 2024). 3Rtau and 4Rtau-tg mice respectively expressed 3Rtau and 4Rtau as expected (Fig. 8A,B). More protein was detected in homogenates from the cortex compared to the hippocampus and more at 9-months of age compared to 3-months of age (Fig. 8A,B). Interestingly, we observed greater 4Rtau accumulation than 3Rtau in either single or double transgenic mice. This was confirmed when using the total tau antibody (Tau5) which again showed greater accumulation of tau in the cortex than in the hippocampus, in an age dependent fashion; however, there did not appear to be an additive effect of expression of both tau transgenes in the 3R/4Rtau-tg mouse compared to either single transgenic line. We also again observed greater accumulation of 4Rtau compared to 3Rtau (Fig. 8A,B). This is in contrast to results observed by immunohistochemistry where we observed similar levels of staining with the same antibodies.

Tau phosphorylation occurs at many locations throughout the protein on both 3R and 4R tau with the majority of phosphorylation site located in the Proline-rich domain or the C-terminus (Martin et al., 2013). To characterize the phosphorylation of tau biochemically, we used Western blot and probed blots with the anti-P-tau (Ser202,Thr205) (clone AT8) and anti-P-tau (Thr181) (clone PHF-6) against phosphorylation sites located in the Proline-rich domain of tau. Interestingly, although we observed P-tau staining with the AT8 antibody by immunohistochemistry in all 3 transgenic tau mouse lines, we only detected the Ser202,Thr205 phosphorylation in 4Rtau-tg and 3R/4Rtau-tg mice by Western blot (Fig. 8A,B). This correlated with the *in vitro* phosphorylated recombinant tau where we only observed bands in the lane containing the P-4Rtau and not the P-3Rtau protein. The immunoblot analysis of P-tau Ser202,Thr205 does not correlate with the IHC results for 3Rtau-tg mice suggesting a difference in the antibody recognition of the 3Rtau protein in fixed tissue compared to biochemical analysis by immunoblot.

In contrast, the Thr181 antibody (PHF-6) detected bands in both the 3Rtau-tg and 4Rtau-tg mice (Fig. 8A,B). Both Thr181 phosphorylated 3Rtau and 4Rtau were detected in the 3R/4Rtau-tg mice although the 3Rtau protein was significantly less than the 4Rtau. Finally, we examined tau phosphorylation in the C-terminus at Ser393/Ser404 using the PHF-1 antibody. Similar to phosphorylation at Ser181, we observed bands at the expected size for 3Rtau-tg and 4Rtau-tg although with a significantly reduced signal for 3Rtau in both the single and bigenic mice (Fig. 8A,B). We consistently observed greater protein accumulation in the cortex compared to the hippocampus and greater accumulation at 9-months of age compared to 3-months of age.

To determine if differences in protein accumulation could be accounted for by changes in RNA transcription, we performed qPCR on RNA isolated from both cortex and hippocampus samples from 3-month and 9-month mice. At 3-months of age, no difference was observed between single transgenic and double transgenic mice at the RNA level for either 3Rtau or 4Rtau (Supplemental Fig. 1A). Interestingly, at 9-months of age, we measured a reduction in 3Rtau RNA in both the cortex and hippocampus of 3Rtau-tg mice compared to 3R/4Rtau-tg mice (Supplemental Fig. 1B). A similar reduction in 4Rtau RNA between single and double transgenic mice was not detected.

### 3.4. Behavioral changes observed in tau transgenic mice

**Fig. 9.**
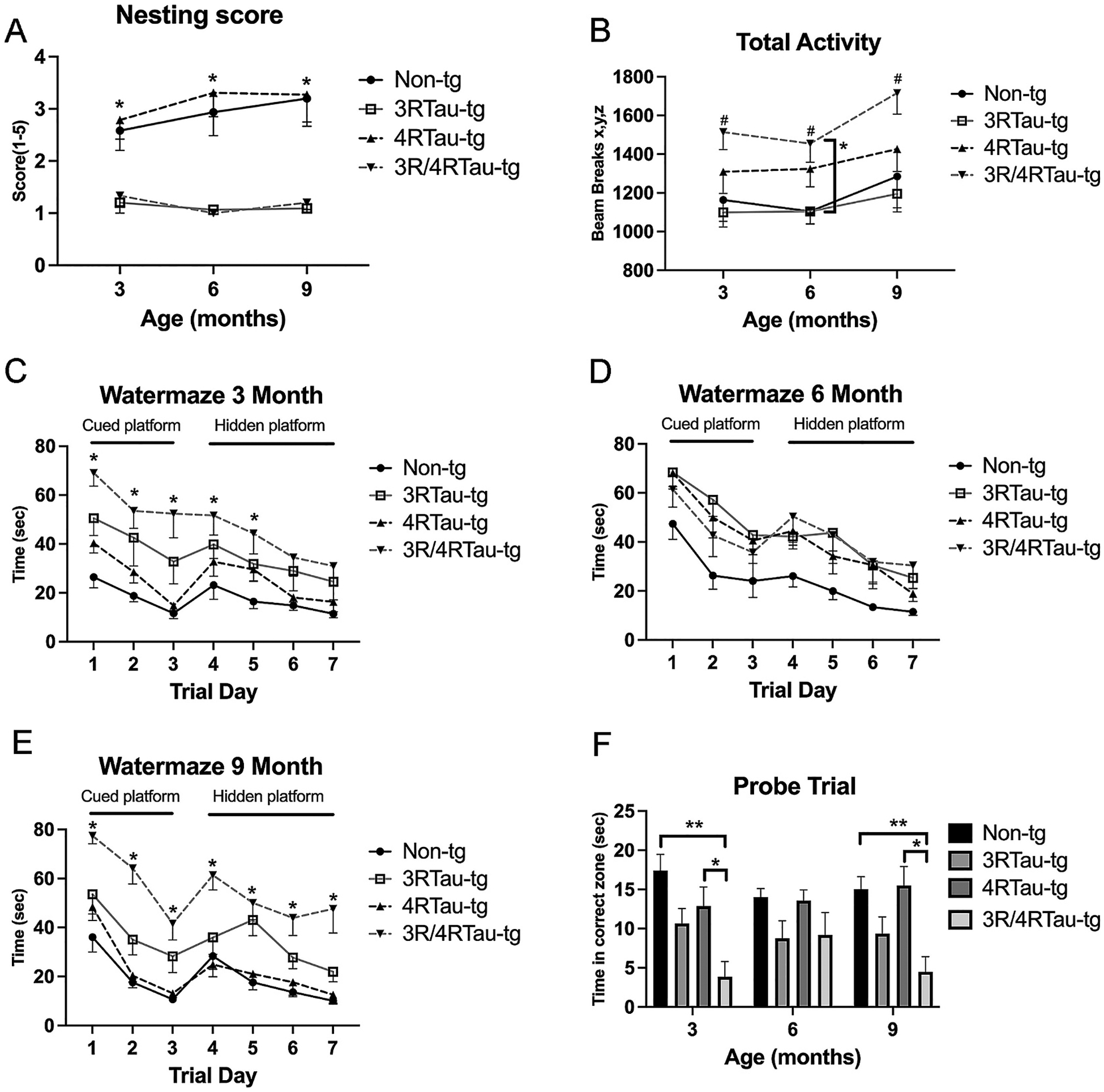
Analysis of behavior alterations in transgenic mice. 3, 6 and 9-month non-tg, 3Rtau-tg, 4Rtau-tg and 3R/4Rtau-tg were assessed for motor coordination and learning and memory. (A) Nest building was scored on a scale of 1–5 with a 5 meaning ideal nest building. (B) Spontaneous motor activity was measured as beam breaks in a monitored cage. Performance in the water maze was measured as time to platform during the cued and hidden (submerged) platform at (C) 3-month, (D) 6-month and (E) 9-month ages. (F) Probe test measured the time spent in the quadrant that previously held the platform. Error bars represent +/− SEM. * - indicates significant difference (A) between both non-tg and 4Rtau groups and 3Rtau and 3R/4Rtau groups; (B, F) between indicated groups (C-E) between non-tg and 4Rtau groups and 3R/4Rtau group (p < 0.05). ** indicates significant difference between indicated groups (*p* < 0.01). N = 10 or more mice per group. Statistical analysis was conducted with two-way ANOVA (mixed model) with Tukey’s test among groups.

All mice exhibit nest building behavior, and changes in this behavior are indicative of depression and/or cognitive deficits such as loss of cortical neurons (Deacon, 2012). In fact, changes in nest building have been characterized in APP-tg mice corresponding to accumulation of Aß (Deacon, 2012). We observed reduced nest building beginning at 3-months and continuing throughout the study in both 3Rtau-tg and 3R/4Rtau-tg mice (Fig. 9A). We did not observe any changes in nest building in 4Rtau-tg mice compared to non-tg mice suggesting that the changes were largely driven by the expression and accumulation of 3Rtau protein.

Our previous characterization of the 3Rtau-tg mice indicated hyperactivity as early as 3-months of age when compared to non-tg littermates (Rockenstein et al., 2015). To determine whether this behavior exists in the bigenic 3R/4Rtau-tg mice, we analyzed the spontaneous activity of the mice over 4 days (Fig. 9B). While we did not observe increased activity in the 3Rtau-tg mice, the 4Rtau-tg mice showed a trend toward increase in activity beginning at 3-months of age. 3R/4Rtau-tg mice had significantly greater spontaneous activity compared to 3Rtau-tg or non-tg mice beginning at 3-months of age and continuing through 9-months of age.

Many models of AD show reduced spatial learning and memory as measured in the Morris water maze. For example, both 3Rtau-tg and 4Rtau-tg mice have deficits in learning and memory when measured with Morris water maze (Ramsden et al., 2005; Rockenstein et al., 2015). To determine if the 3R/4Rtau-tg mice also had memory impairments, all 3 transgenic lines and non-tg mice were assessed by Morris water maze at 3, 6 and 9-months (Fig. 9C–E). Beginning at 3-months of age and then persisting through 9-months of age, the 3R/4Rtau-tg mice consistently performed significantly worse at the water maze compared to non-tg mice. The 3Rtau-tg mice performance was intermediate between the 3R/4Rtau and the non-tg. No significant impairment was measured in learning and memory for the 4Rtau-tg mice. Following the 7-day water maze assessment, we removed the platform and measured the time mice spent in the quadrant that previously contained the platform (Probe trial, Fig. 9F). 3R/4Rtau-tg mice performed significantly worse in this task compared to non-tg or 4Rtau-tg mice at 3 and 9-months. 3Rtau-tg mice again performed intermediately between the 3R/4Rtau-tg and non-tg mice. These data suggests that additional over-expression/ accumulation of 4Rtau, while on its own does not significantly affect learning and memory, in combination with 3Rtau can significantly impair learning and memory.

## 4. Discussion

In this study, we developed and characterized a novel mouse model of tauopathy overexpressing both human 3-repeat and 4-repeat tau isoforms. We found that overexpression of both tau isoforms results in significantly greater tau accumulation and neuroinflammation compared to overexpression of either isoform alone. This increased pathology was associated with significant deficits in 3R/4Rtau-tg mice in total activity and learning and memory when compared to either 3Rtau-tg or 4Rtau-tg alone. Interestingly, measurement of nesting behavior showed similar deficits for both 3Rtau-tg and 3R/3Rtau-tg but not the 4Rtau-tg mice. Thus, accumulation of both 3Rtau and 4Rtau results in neuronal and behavioral impairments that are specific to the expression of each tau isoforms resulting in a unique mouse model of tauopathy and a more complete representation of human tauopathy.

### 4.1. Tau isoforms, neuropathology and cognitive decline

Previous characterization of overexpression of 3Rtau in a mouse model of Pick’s disease by immunohistochemistry demonstrated increased total tau and P-tau (Ser202/Thr205) accumulation in the cortex and dentate gyrus of the hippocampus (Rockenstein et al., 2015). We confirmed and expanded on these findings by examining the CA1 and CA3 of the hippocampus and observed both neuronal soma and neurite accumulation that extends deep in the parenchyma of the hippocampus. While in CA1, we observed 3Rtau accumulation in nearly all neurons, we observed less accumulation in CA3 neurons and accumulation in interneurons of the CA3. This difference may be due to expression of tau directly in these cells or could be caused by cell-to-cell spread of tau from neurons in the dentate gyrus where we see accumulation of tau all along the suprapyramidal mossy fibers leading to the CA3. Pathological tau tangles occur intra-neuronally, and recent evidence suggests the possibility of cell-to-cell propagation (Simic et al., 2016). Tau oligomers or aggregates injected into wild-type or tau transgenic mice propagate from the site of injection and *in vitro* cultures show propagation of tau from cell-to-cell (Simic et al., 2016). Recently, we and others have detected tau in exosomes in patients with AD and other tauopathies suggesting this might be a mechanism for cell-to-cell propagation (Saman et al., 2012; Shi et al., 2016; Winston et al., 2016; Woerman et al., 2016).

We further expanded on the characterization of P-tau by Western blot for tau phosphorylation sites in both the proline rich domain and the C-terminus of tau. Western blot for phosphorylated tau in homogenates from the cortex and hippocampus using antibodies against P-tau (Ser396/Ser404), P-tau (Ser202/Thr205) as well as P-tau (Thr181) showed an interesting pattern of phosphorylation of the 3R and 4R tau proteins. Phosphorylation at Ser202/Thr205 was exclusively observed in the lysates from the 4Rtau-tg and 3R/4Rtau-tg mice but not from the 3Rtau-tg mice even though we observed positive staining by immunohistochemistry with the same antibody. *In vitro* phosphorylated 3Rtau and 4Rtau confirmed these observations where we only saw P-4Rtau signal. This contrasted with P-tau Ser393/Ser404 at the C-terminus and P-tau Thr181 in the proline rich domain where we observed signal at both 3Rtau and 4Rtau as previously observed for another 3Rtau-tg mouse (Higuchi et al., 2002). P-tau Thr181 has been used as plasma biomarker for AD (Meng and Lei, 2020) and the P-tau Ser393/Ser404 has been linked to synaptic loss and intracellular tangles in mouse models of AD and human AD and Down syndrome patients (Mondragon-Rodriguez et al., 2014; Santa-Maria et al., 2012; Torres et al., 2021). Thus, the 3R/4Rtau-tg mouse model accumulated tau that was phosphorylated at multiple locations in the proline-rich domain as well as the C-terminus domain similar to AD patients.

Interestingly, overexpression of 4Rtau in the bigenic mouse had little impact on the accumulation of total tau or P-tau (Ser202/Thr205) in the dentate gyrus or CA1, suggesting 3Rtau accumulation drives tau accumulation in both regions. As previously reported, we observed neuronal loss in the hippocampus of the 4Rtau-tg mouse (Yoshiyama et al., 2007), which was exacerbated in the bigenic mouse. Previous characterization of the 4Rtau-tg mouse described increased astrocytosis in the hippocampus by 9-months of age (Yoshiyama et al., 2007). Significant hippocampal astrocytosis and microglial activation occurred in the bigenic mouse compared to 3Rtau-tg or 4Rtau-tg mice, as early as 3-months of age, suggesting that the expression of both isoforms of tau enhance the inflammatory response in the CNS.

As previously reported, the 3Rtau-tg (Rockenstein et al., 2015) and 4Rtau-tg (Saito et al., 2019) showed significant deficits in learning and memory when measured with the Morris water maze. Although we observed reduced learning and memory for these single transgenic mice, we only observed statistically significant deficits with the bigenic 3R/4Rtau-tg mice. Similarly, for total activity, although previous investigation has shown a hyperactivity for the 3Rtau-tg mouse (Rockenstein et al., 2015), we only observed this in the bigenic 3R/4Rtau-tg mouse. Mice exhibit reduced nest building due to cognitive deficits and/or depression (Deacon, 2012). Cognitive deficits and even depression have been noted for Alzheimer’s patients (Lyketsos and Olin, 2002). We observed reduced nest building in both the 3Rtau-tg and the 3R/4Rtau-tg mice that was significantly different from both the 4Rtau-tg and non-tg mice. In this instance, we believe that the over-expression and accumulation of 3Rtau is the driving factor for the reduced nest building.

### 4.2. Relationships between tau isoforms and inflammation

Neural inflammation has been extensively studied in the context of several neurodegenerative diseases (Bennett and Viaene, 2021; Bruck et al., 2016). In particular, astrocytes closely associate with Aß plaques, and reactive microglial cells are increased both in post-mortem human AD patient brains and AD mouse models (Funato et al., 1998; Nagele et al., 2003). We observed increased astrocytosis in the cortex of both 3Rtau and the bigenic 3R/4Rtau mice. The accumulation was greater for the bigenic mouse suggesting expression of 4Rtau exacerbates the inflammatory response observed only when 3Rtau is overexpressed. Interestingly, we did not observe increased astrocytosis in the 4Rtau-tg mouse until 9-months of age. Similarly, we observed increased activation of microglia in both the cortex and hippocampus when either 3Rtau or 4Rtau was expressed. This was significantly increased when both tau proteins were expressed in the bigenic mouse. Thus, both 3Rtau and 4Rtau are contributing to the inflammatory response and may be acting synergistically.

### 4.3. Differential pathological patterns and translatability to human tauopathy

It is interesting that the expression 3Rtau and 4Rtau are not uniformly expressed in all cell types examined. That is, although the two isoforms appear to be expressed equivalently in cortical neurons, 3Rtau was preferentially expressed in the CA1 of the hippocampus with accumulation in the neurites. In contrast, 4Rtau was preferentially expressed in the CA3, and with limited CA1 accumulation in the soma and sparse expression in the neurites. This may be due to the different transgenic promoters driving expression of the 3Rtau and 4Rtau transgenes with 3Rtau expressed from the mThy-1 promoter (Rockenstein et al., 2015) and 4Rtau expressed from the MoPrP promoter (Yoshiyama et al., 2007). Addition of both tau transgenes in the double transgenic (3R/4Rtau-tg) mice did not appear to significantly increase the accumulation of T-tau or P-tau compared to single transgenic mice as determined by IHC (Figs. 3,4) or immunoblot (Fig. 8). It is important to note that the expression of both human 4Rtau and 3Rtau in these models occurs on the background of endogenous mouse tau expression. Mice express 0N3Rtau predominantly at birth until about P30, then 4Rtau predominates expression (Hernandez et al., 2020). So, the 3R/4Rtau-tg mice characterized in this study overexpress human 3Rtau and 4Rtau but also express the endogenous mouse 4Rtau.

We noted variability among similarly aged mice that shared the same genotype. The 3Rtau-tg mouse is on the DBA background and the 4Rtau-tg mouse is on the B6C3H/F1 background. Since the crosses of these mice resulting in the 3R/4Rtau-tg mice were not backcrossed onto a single background, there would be variability in background of the F1 generation. This variability may be beneficial as a model of AD modeling the diverse background in the human population. Furthermore, a recent transcriptomic analysis of transposable elements (TE) in 3Rtau, 4Rtau and 3R/4Rtau overexpressing immortalized human neurons found expression patterns in 3R/4Rtau expressing neurons more closely mimic the TE expression patterns in human AD than either tau isoform alone (Grundman et al., 2021). Thus, a mouse model expressing both 3Rtau and 4Rtau could better emulate gene expression alterations observed in human AD and could provide a better model for testing tau based therapeutic options.

Transgenic mouse models overexpressing the 4Rtau protein show increased tau accumulation in the cortex and hippocampus in a promoter dependent manner (Sahara and Yanai, 2023). The isoform of tau expressed in the 4Rtau-tg mouse used in this study, P301L/S, has been associated with late onset AD and these models generally recapitulate many of the aspects of AD; however, they lack the parallel expression of 3Rtau observed in human AD. The addition of the 3Rtau-tg mouse with the L266V and G272V mutation associated with FTD and Pick’s disease may accelerate the AD pathology in the double transgenic mouse. Although this may complicate long-term studies, this allows the use of younger mice to study therapies associated with reducing tau accumulation and thus reducing costs associated with testing.

Mouse transgenic models of tauopathy containing the full human tau gene expressing both 3R and 4Rtau have been generated previously (Duff et al., 2000; Sahara and Yanai, 2023; Saito et al., 2019) and even expressed on a mouse tau knockout background (Andorfer et al., 2003). While these models express 3Rtau and 4Rtau; 3Rtau is expressed at a greater level possibly making this a better model of Pick’s disease which is characterized by accumulation of 3Rtau (Sahara and Yanai, 2023). To correct for the inherent 3Rtau bias in these models, He et al. crossed a mouse containing the full human tau gene with the 4Rtau transgenic mouse to generate additional expression of 4Rtau (He et al., 2020). This mouse has an approximate 1:1 ratio of 3R:4tau similar to human tau expression levels in AD. However, none of these models have demonstrated cell-to-cell spread of tau in the hippocampus, and only Andorfer et al. generated model (huTau on murine tau knockout background) showed a deficit in learning and memory, food burrowing and object discrimination (Andorfer et al., 2003; Geiszler et al., 2016; Phillips et al., 2011; Polydoro et al., 2009).

## 5. Conclusions

Immunotherapy approaches for the reduction of tau in AD have focused on both active and passive immunization. Active immunization protocols utilizing a peptide of tau can elicit a generalized immune response against all tau species (Pedersen and Sigurdsson, 2015; Valera et al., 2016), and passive immunization strategies developed at targeting either total 4Rtau protein or phosphorylated or acetylated tau have been examined (Pedersen and Sigurdsson, 2015; Valera et al., 2016). In fact, tau immunotherapy in the APP-tg mouse model of AD showed reduction in not only total tau, but also Aß oligomers suggesting targeting tau may have beneficial downstream effects (Castillo-Carranza et al., 2015). However, without the appropriate model of tauopathy, one must wonder if the effects observed with these therapeutic approaches can be reflected in patients with AD where both isoforms of tau are equally expressed. Therefore, this model of 3R/4Rtau may better reflect the environment of AD tauopathy and thus may be a better mouse to test novel tau therapeutics.

## Supplementary Material

MMC6

MMC5

MMC3

MMC4

MMC1

MMC2

MMC7

MMC8

Supplementary data to this article can be found online at https://doi.org/10.1016/j.nbd.2025.106942.

## Data Availability

Data will be made available on request.
