# Supplementary figures and images for: Differential roles of human tau isoforms in the modulation of inflammation and development of neuropathology
Source: Neurobiol Dis. Author manuscript; Available in PMC 2025 Jul 1. (PMC12178306; doi:10.1016/j.nbd.2025.106942)

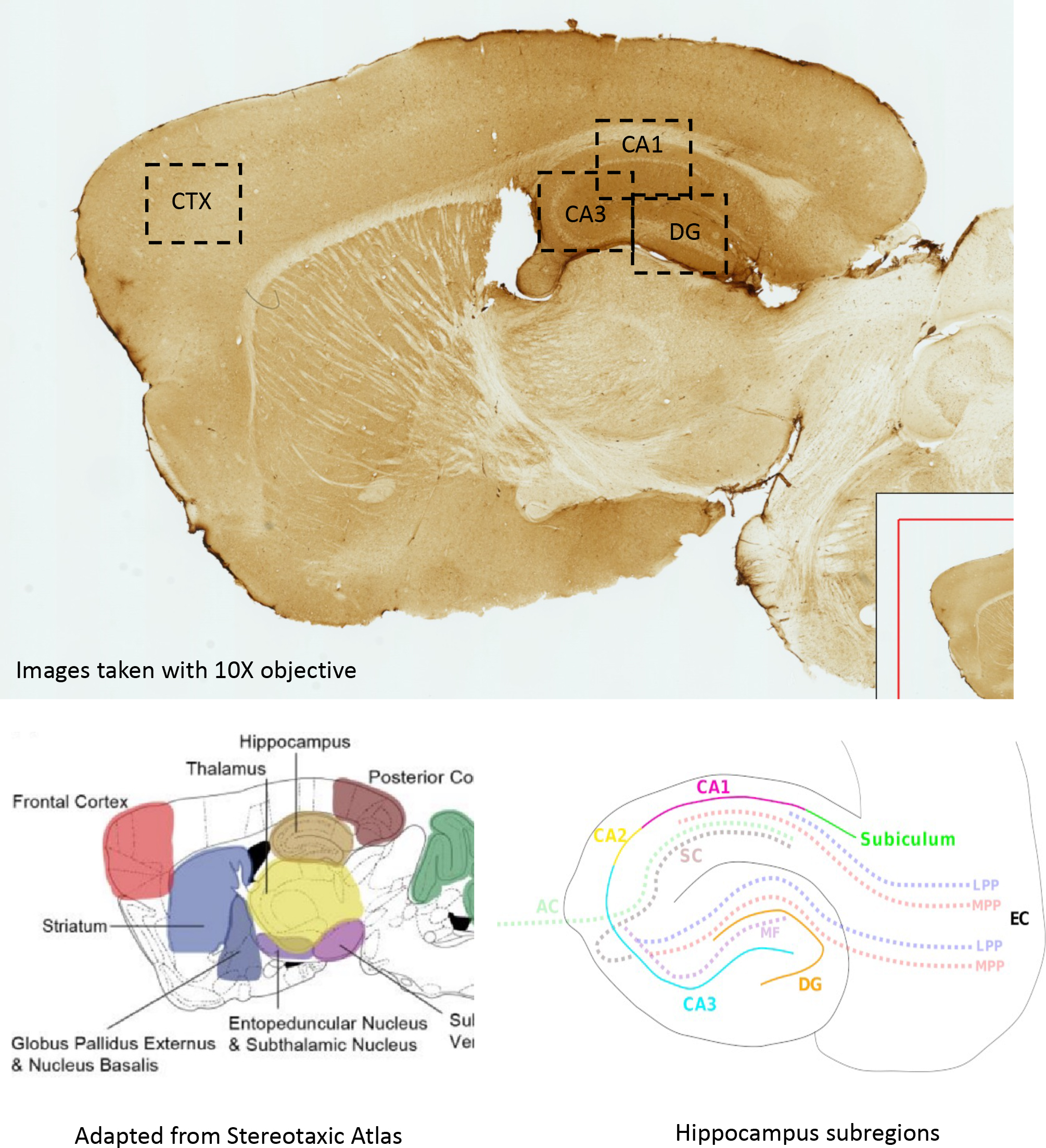

Supplement: MMC6 [file NIHMS2083368-supplement-MMC6.jpg]

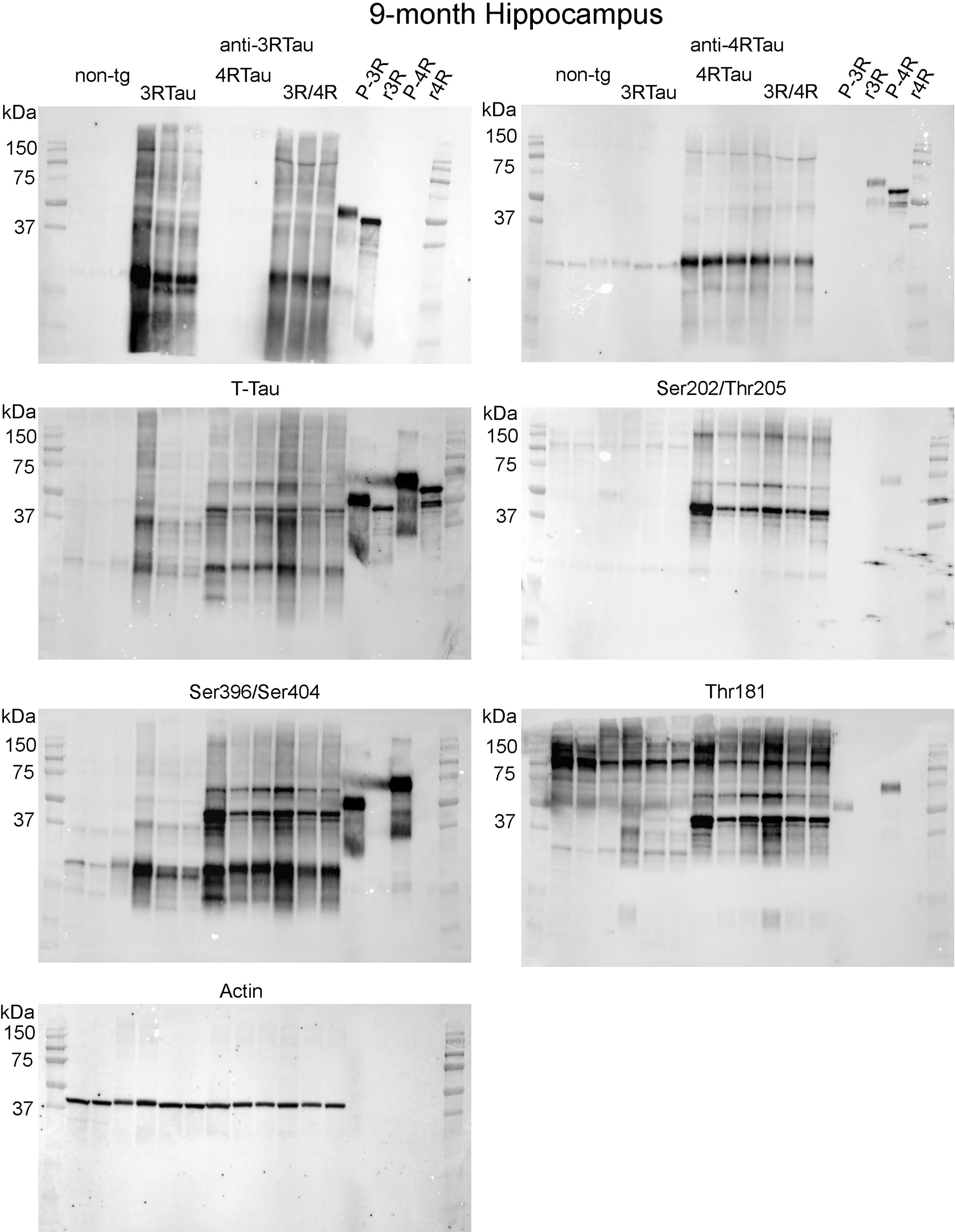

Supplement: MMC5 [file NIHMS2083368-supplement-MMC5.jpg]

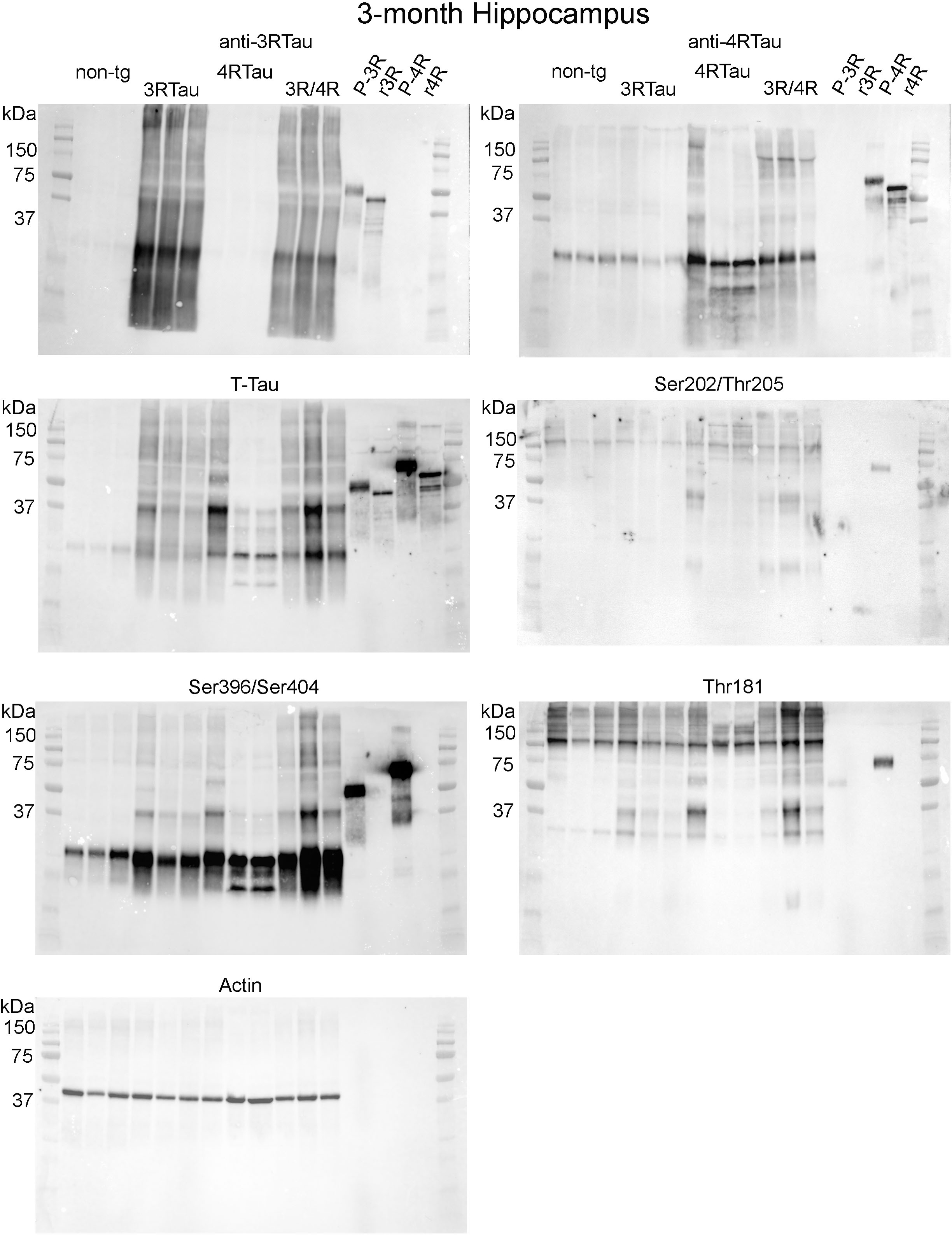

Supplement: MMC3 [file NIHMS2083368-supplement-MMC3.jpg]

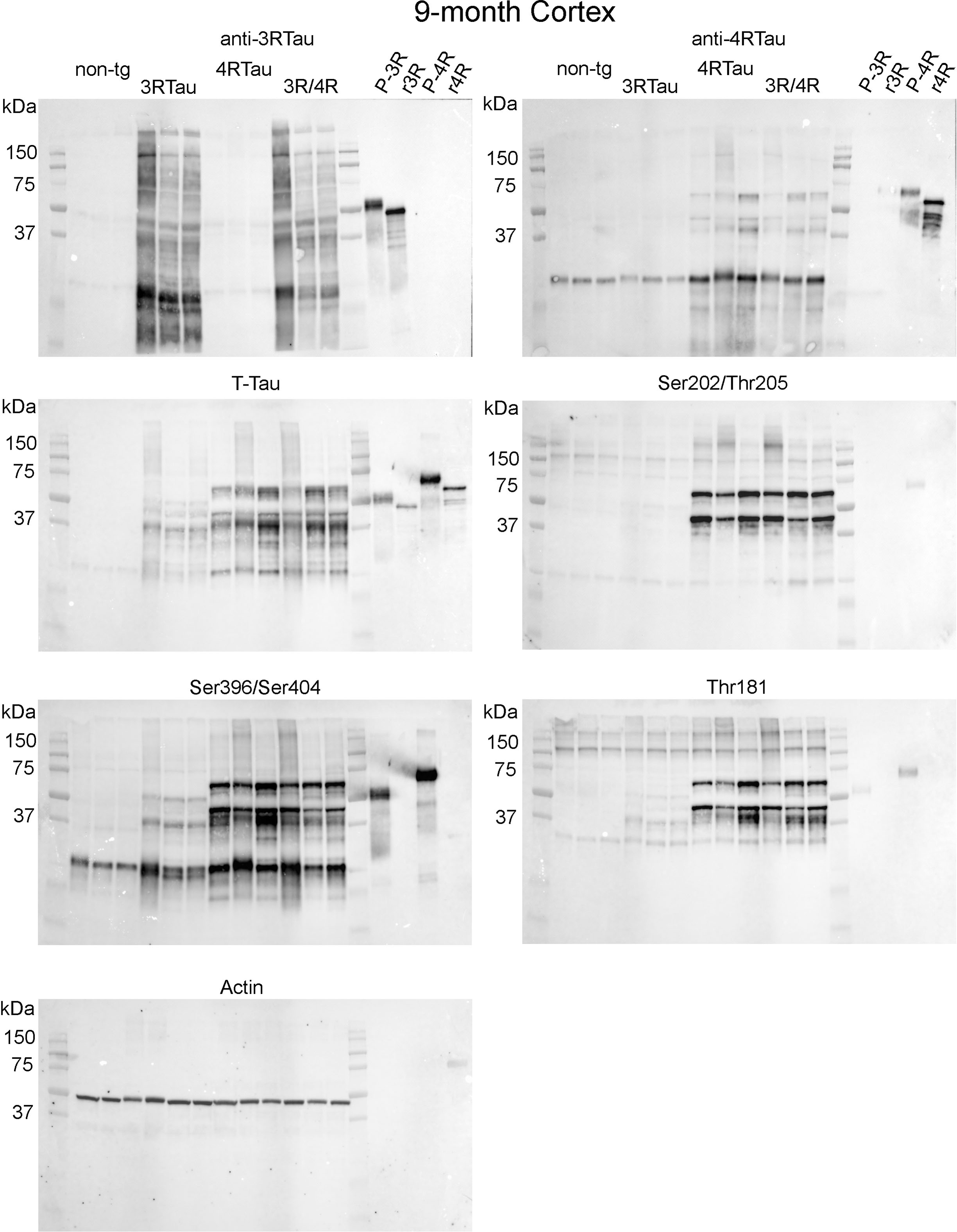

Supplement: MMC4 [file NIHMS2083368-supplement-MMC4.jpg]

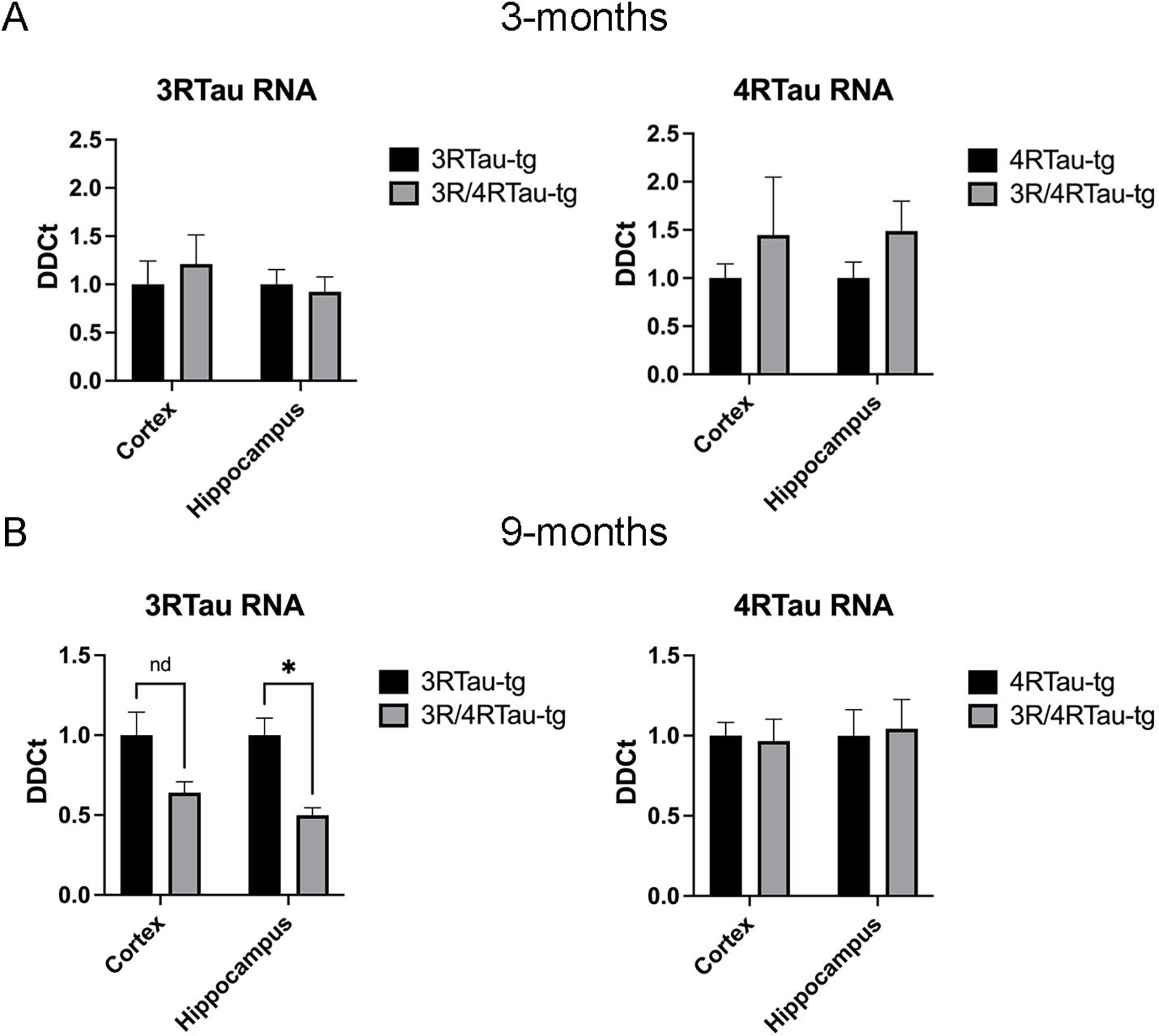

Supplement: MMC1 [file NIHMS2083368-supplement-MMC1.jpg]

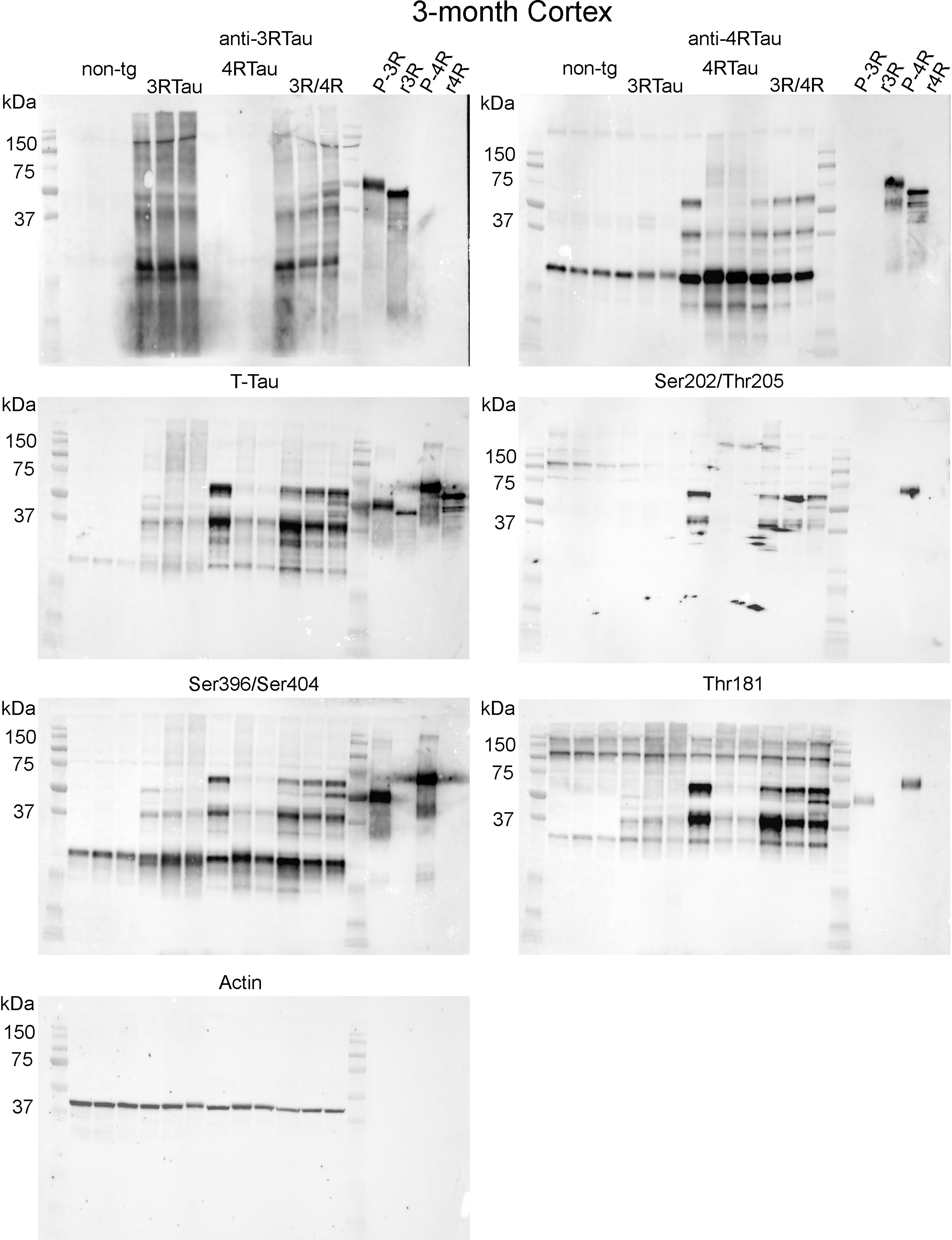

Supplement: MMC2 [file NIHMS2083368-supplement-MMC2.jpg]

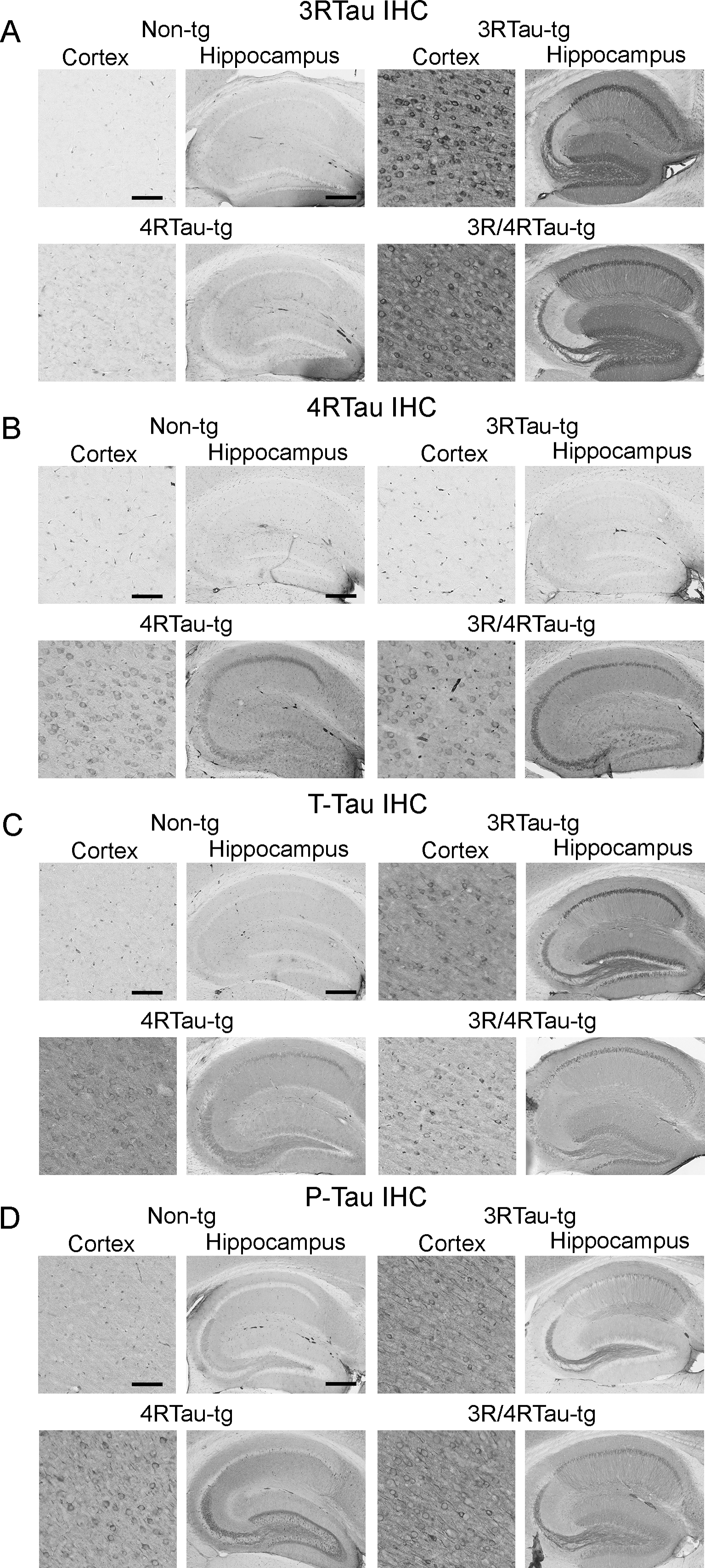

Supplement: MMC7 [file NIHMS2083368-supplement-MMC7.jpg]

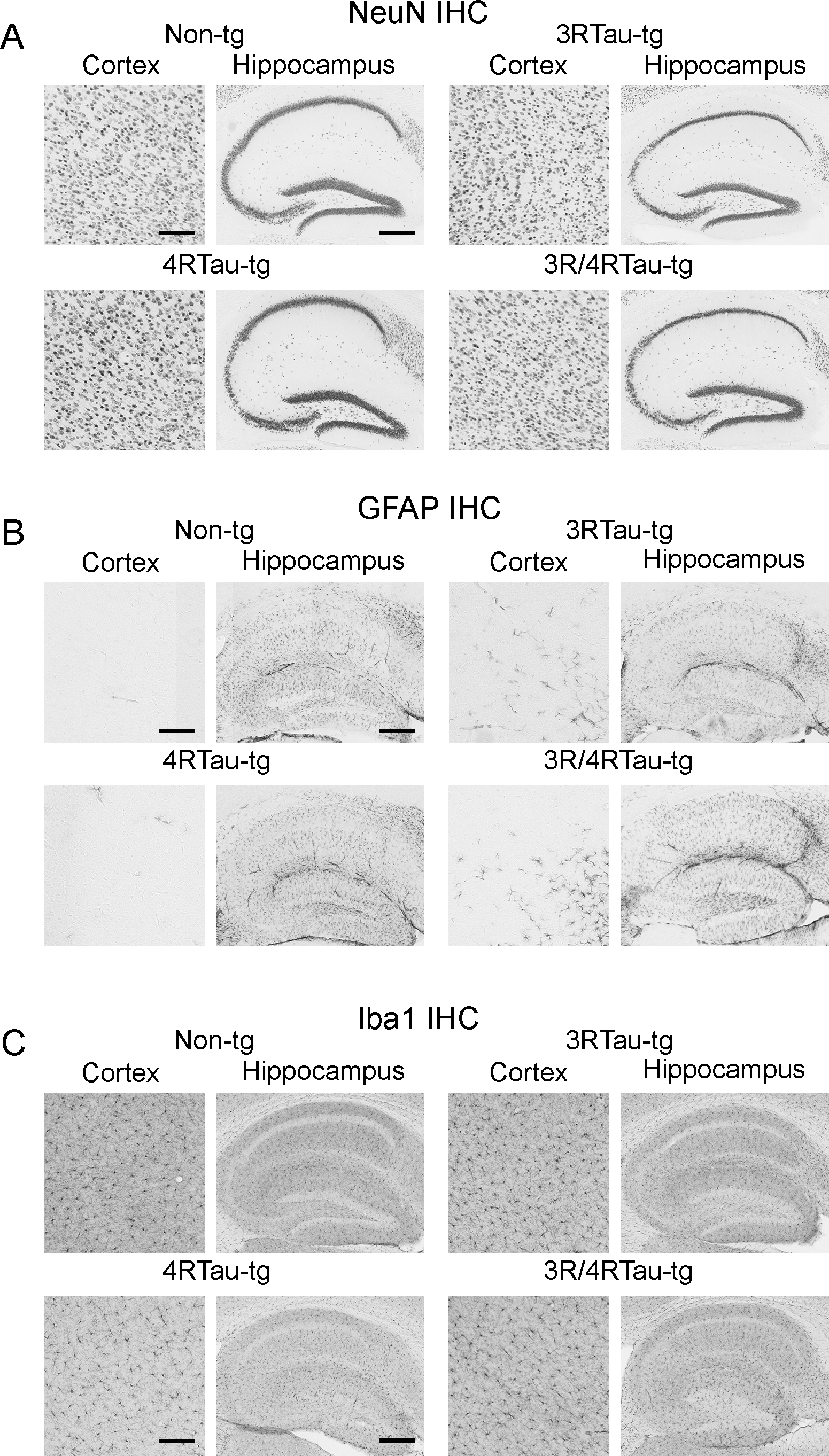

Supplement: MMC8 [file NIHMS2083368-supplement-MMC8.jpg]
